# *DMDRMR* promotes angiogenesis via antagonizing DAB2IP in clear cell renal cell carcinoma

**DOI:** 10.1038/s41419-022-04898-3

**Published:** 2022-05-13

**Authors:** Yumeng Zhu, Xiaojun Liu, Yang Wang, Yongbo Pan, Xiaoqi Han, Bo Peng, Xu Zhang, Shaoxi Niu, He Wang, Qinong Ye, Yinmin Gu, Shan Gao

**Affiliations:** 1 School of Biomedical Engineering (Suzhou), Division of Life Sciences and Medicine, University of Science and Technology of China, 230026 Hefei, China; 2 CAS Key Laboratory of Bio-medical Diagnostics, Suzhou Institute of Biomedical Engineering and Technology, Chinese Academy of Sciences, 215163 Suzhou, China; 3 Shanxi Academy of Advanced Research and Innovation, 030032 Taiyuan, China; 4 Medical College, Guizhou University, 550025 Guiyang, China; 5 Department of Urology, The First Medical Center of Chinese PLA General hospital, 100853 Beijing, China; 6 Department of Medical Molecular Biology, Beijing Institute of Biotechnology, Collaborative Innovation Center for Cancer Medicine, 100850 Beijing, China; 7 School of Medicine, Southeast University, 210096 Nanjing, Jiangsu China; 8 Zhongda Hospital, School of Life Sciences and Technology, Advanced Institute for Life and Health, Southeast University 210096 Nanjing, China

**Keywords:** Tumour angiogenesis, Tumour biomarkers

## Abstract

Clear cell renal cell carcinoma (ccRCC) patients are highly angiogenic and treated by targeted therapies against VEGFA/VEGFR signaling pathway. However, tumors with such targeted therapies remain a significant clinic challenge. Understanding the underlying mechanism against angiogenesis is highly desired. Here, we demonstrated that the lncRNA *DMDRMR* serves as a sponge of miR-378a-5p to increase EZH2 and SMURF1 expression, thus promoting EZH2-mediated transcriptional repression of DAB2IP and SMURF1-mediated degradation of DAB2IP. Consequently, this axis activates VEGFA/VEGFR2 signaling pathway, resulting in angiogenesis and resistance of tumor cells to sunitinib in ccRCC. Moreover, the competing endogenous RNA regulatory axis of *DMDRMR* is clinically relevant to ccRCC pathogenesis and prognosis of patients with ccRCC. Our results support that the *DMDRMR*/miR-378a-5p/DAB2IP axis may serve as a novel target for combination diagnosis or therapy of ccRCC patients. Our findings may have highly clinical relevance for future translation to develop the targeted therapies for patients with ccRCC.

## Introduction

Clear cell renal cell carcinoma (ccRCC) is a major type of RCCs and characterized by high angiogenesis and dense vascularization [1, 2]. A common genetic mutation in ccRCC is loss of the *von Hippel-Lindau* gene, which results in stabilization of hypoxia-inducible factors (HIFs), and contributes to the activation of HIF target genes, including vascular endothelial growth factor (VEGF) [3]. The deregulated VEGFA/VEGF receptor (VEGFR) signaling pathway represents an ideal therapeutic target for advanced ccRCC treatment. However, most patients acquire drug resistance with the targeted angiogenesis therapy [3]. Therefore, further understanding of tumor angiogenesis is highly desired.

Long non-coding RNAs (lncRNAs) with a minimum 200 bases in length have been shown to regulate gene expression in multiple layers, including transcription, translation, and post-transcriptional/translational modification through multiple ways, such as binding regulatory proteins and acting as microRNA (miRNA) sponges [4, 5]. Emerging evidence indicates that lncRNAs may act as new modulators in angiogenesis [6], which need to be further dissected.

In this study, we expanded the function of our previously reported *DMDRMR* [7] and revealed that *DMDRMR* activates the VEGFA/VEGFR2 signaling pathway by sponging miR-378a-5p to promote EZH2 and SMURF1-mediated repression of DAB2IP expression, resulting in enhanced angiogenesis and sunitinib resistance. These findings further highlight the key roles of *DMDRMR* in ccRCC.

## Materials and methods

### Cell culture

The human umbilical vein endothelial cells (HUVECs) were obtained from Prof. YF Zhou (Soochow University) and cultured in HUVECs specialized medium (Procell, CM-0122). The human embryonic kidney HEK293T (293T) cell line was cultured in DMEM medium (Gibco) supplemented with 10% fetal bovine serum (FBS). 786-O and 769-P cell lines were maintained in RPMI-1640 medium (Gibco) supplemented with 10% FBS. 293T, 786-O, and 769-P cell lines were purchased from the Shanghai Cell Bank Type Culture Collection Committee (Shanghai, China). All these cells were maintained in a 37 °C incubator in a humidified atmosphere containing 5% CO_2_ and were previously examined negative for mycoplasma contamination.

### Enzyme-linked immuno sorbent assay

The concentration of VEGFA in the supernatants of cell cultures were measured using Human VEGFA ELISA Kit (ABclonal Technology, RK00023) according to the guidelines of the manufacturer. Briefly, 100 μl/well of standard and test samples were loaded into 96-well plates. After incubating with biotin-conjugate antibody, streptavidin-conjugated horseradish peroxidase (HRP) was added to each well and reacted with the HRP substrate solution. Detect the optical density within 5 minutes (min) under 450 nm and correct the wavelength set at 570 nm.

### Matrigel tube formation assay

In all, 10 μl of matrigel (Corning Inc., NY, USA) was thawed on ice at 4 °C overnight, added into each well of a precooled μ-Slide Angiogenesis plate (Ibidi), and incubated at 37 °C for 30 min for hardening. In total, 1 × 10^4^ HUVECs in 100 μl conditioned cell culture medium were plated onto the precoated matrigel for 24 hours (h). The resulting capillary-like structures in each well were then photographed with a microscope and counted with ImageJ software.

### In vivo matrigel plug angiogenesis assay

The conditioned cell medium from *DMDRMR* KD cells was collected and concentrated, 300 μl of concentrated medium mixed with 400 μl of BD Matrigel™aBasement Membrane Matrix and 1 × 10^7^ HUVECs were injected subcutaneously into the back of the 5-week old nude male mice. After 10 days, the skin was pulled back with scissors to expose intact Matrigel plugs, and plug images were taken. The hemoglobin content of the matrigel plugs were determined using Hemoglobin Assay kit (Abcam, ab234046). Histological section on slides were stained with hematoxylin and eosin (H&E). All protocols involving animals were previously approved by the Ethics Committee for the Use of Experimental Animals of the Suzhou Institute of Biomedical Engineering and Technology, Chinese Academy of Sciences (Suzhou, Jiangsu, China).

### Statistical analyses

All data were presented as mean ± standard deviation (SD) or mean ± standard error of the mean (SEM). All experiments with statistical analysis have been repeated at least three times. Two-tailed Student’s *t* test was performed to analyze the difference between two groups. A two-sided *χ*^2^ test was used to assess the statistical significance of the association between the expression of RNA or protein levels and the clinico-pathological parameters of patients. For the kaplan–meier survival analysis, a log-rank test was performed. For the correlation analysis, spearman’s correlation was performed. “pROC” package in R software was used to construct receiver operating characteristic (ROC) curves and then to calculate area under curve (AUC). All statistical analyses were performed using GraphPad Prism 8.0 (GraphPad software, Inc.) or R software (version 3.5.2). A value of *p* < 0.05 was considered statistically significant difference.

Additional methods can be found in the Supplementary Material and Methods.

## Results

### *DMDRMR* Induces VEGFA production

**Fig. 1:**
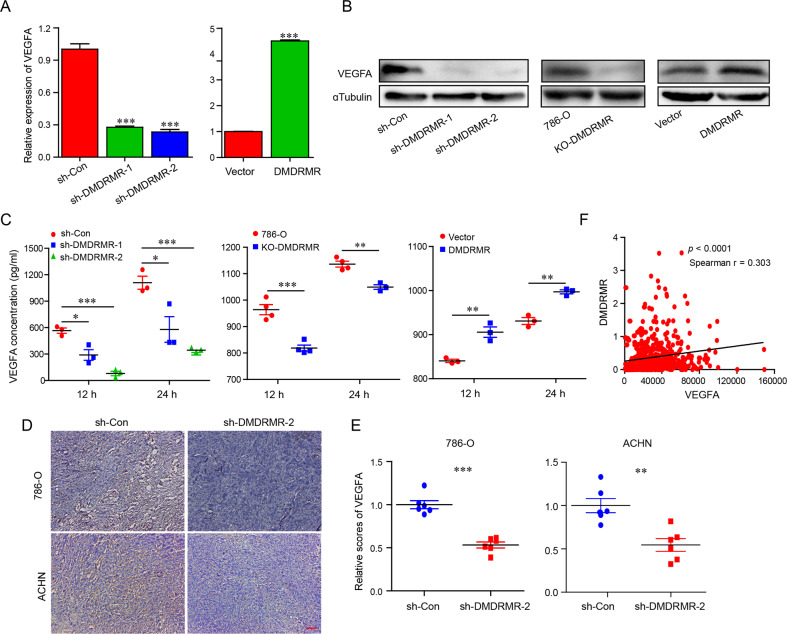
*DMDRMR* increases VEGFA production. **A** The qRT-PCR analysis of *VEGFA* in *DMDRMR* KD 786-O cells (left) and *DMDRMR* OE 769-P cells (right). Sh-Con/vector represents a negative control of short-hairpin/empty vector plasmid. **B** Immunoblot of VEGFA in *DMDRMR* KD (left) and KO (middle) 786-O cells and OE 769-P (right) cells. **C** ELISA assays detecting the secreted VEGFA levels in *DMDRMR* KD (left) and KO (middle) 786-O cells and OE 769-P (right) cells in the indicated times. **D**, **E** Representative bright-field images (**D**) and quantification (**E**) of VEGFA IHC staining from tumor tissues of recipient NOD/SCID/IL-2Rγ-null (NSG) mice injected with *DMDRMR* KD 786-O and ACHN cells (scale bars, 200 µm; *n* = 6). **F** Correlation analysis between VEGFA and *DMDRMR* expression levels in TCGA ccRCC and adjacent tissues. The results are presented as mean ± SEM. *p < 0.05, **p < 0.01, ***p < 0.001.

In our previous study, the RNA-sequence and quantitative real-time polymerase chain reaction (qRT-PCR) data from *DMDRMR* knockdown (KD) and control 786-O cells showed that KD of *DMDRMR* decreases the transcriptional level of *VEGFA* [7]. Here, we further confirmed the effects of *DMDRMR* on VEGFA expression. qRT-PCR analyses showed that KD of *DMDRMR* decreased and overexpression (OE) of *DMDRMR* increased the transcriptional level of *VEGFA* (Figs. 1A and S1A, B). Immunoblot and enzyme-linked immuno sorbent assay (ELISA) assays confirmed that both KD and knockout (KO) of *DMDRMR* reduced, whereas OE of *DMDRMR* upregulated protein and secreted levels of VEGFA (Fig. 1B, C). In our previous mouse xenograft models [7], *DMDRMR* KD reduced VEGFA expression levels (Fig. 1D, E). Consistently, *DMDRMR* expression level was positively correlated with *VEGFA* expression level in The Cancer Genome Atlas (TCGA) ccRCC cohort (Fig. 1F). These results suggest that *DMDRMR* increases VEGFA expression and induces VEGFA production.

### *DMDRMR* drives angiogenesis

**Fig. 2:**
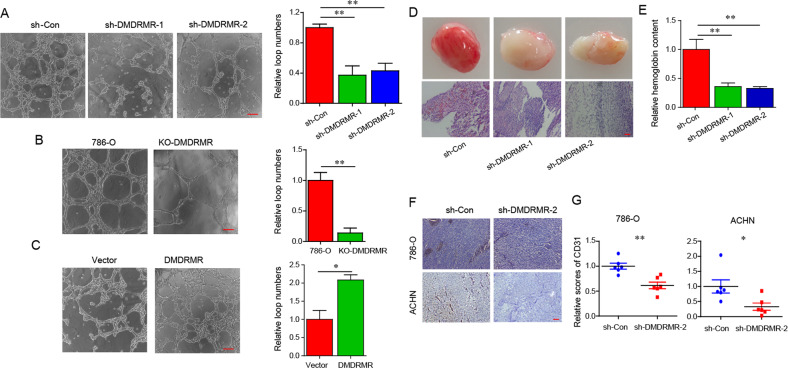
*DMDRMR* drives tumor angiogenesis. **A**–**C** Representative bright-field images (left) and quantification (right) of matrigel tube formation of HUVECs incubated with conditioned medium from *DMDRMR* KD (**A**) and KO (**B**) 786-O cells and OE 769-P (**C**) cells (scale bars, 200 µm). **D** Representative images (top) and H&E staining (bottom) of subcutaneous matrigel plug assay in *DMDRMR* KD mice (scale bars, 200 µm). **E** Relative hemoglobin contents were measured from the experiment (*n* = 6). **F**, **G** Representative bright-field images (**F**) and quantification (**G**) of CD31 IHC staining from tumor tissues of NSG mice injected with *DMDRMR* KD 786-O and ACHN cells (scale bars, 200 µm; *n* = 6). The results are presented as mean ± SEM. *p < 0.05, **p < 0.01, ***p < 0.001.

Given that VEGFA is an inducer of angiogenesis and endothelial cell tube formation [8], we wondered whether *DMDRMR* regulates angiogenesis in ccRCC. Firstly, we performed gene ontology (GO) pathway analysis and found that 1039 differentially expressed genes (DEGs) from *DMDRMR* KD and control 786-O cells [7] were enriched for angiogenesis-related pathways, such as angiogenesis, sprouting angiogenesis, and regulation of VEGF production (*FDR* < 0.05) (Fig. S1C, D). Consistently, based on the median value of *DMDRMR* expression in ccRCC patients from TCGA, Gene Set Enrichment Analysis (GSEA) revealed that *DMDRMR* expression is associated with VEGF-related pathways including positive regulation of VEGF production and VEGF pathway (*FDR* < 0.25) (Fig. S1E, F), suggesting that *DMDRMR* might be an important modulator of angiogenesis in ccRCC. To functionally validate these findings, we evaluated the effect of *DMDRMR* expression on in vitro angiogenesis activities of vascular endothelial cells. HUVECs were incubated with conditional medium collected from ccRCC cells expressing variable amounts of *DMDRMR*, and the capillary tube formation of HUVECs were measured. Both *DMDRMR* KD and KO reduced, whereas *DMDRMR* OE increased the amounts of capillary tube formation (Fig. 2A–C). In vivo matrigel plug angiogenesis assay showed that *DMDRMR* KD reduces blood vessel formation as evidenced by decreased redness and less numbers of capillaries (Fig. 2D). Similarly, the amounts of hemoglobin were markedly reduced in *DMDRMR* KD (Fig. 2E). In our previous mouse xenograft models [7], the vascular endothelial cell marker CD31 staining of tumors indicated that *DMDRMR* KD inhibited the generation of microvessels (Fig. 2F, G). Taken together, these results indicate that *DMDRMR* promotes angiogenesis in ccRCC.

### VEGFA is an indirect-regulated gene of *DMDRMR*

To define the molecular mechanisms of *DMDRMR* in driving angiogenesis, we firstly investigated whether *DMDRMR* directly regulates VEGFA expression. Given *DMDRMR* transcriptionally regulates VEGFA expression, we examined whether *DMDRMR* affects the transcription of *VEGFA* or the mRNA stability of *VEGFA* transcript. The nuclear run-on assay showed that the transcription efficiency of *VEGFA* was not significantly altered after *DMDRMR* KD and KO (Fig. S2A, B), which excludes the regulation of *DMDRMR* to *VEGFA* at transcriptional level. We further treated 786-O cells with the transcriptional inhibitor actinomycin D and assessed the mRNA stability of *VEGFA*. The half-lives of *VEGFA* mRNA remained similar in *DMDRMR* KD and KO cells (Fig. S2C, D). Moreover, MS2-based GFP RNA immunoprecipitation (RIP) revealed that *VEGFA* was not enriched for *DMDRMR* (Fig. S2E). Furthermore, it is known that *DMDRMR* regulates VEGFA expression without utilizing its binding protein IGF2BP3 [7]. These data suggest that VEGFA serves as an indirect downstream effector of *DMDRMR* to regulate angiogenesis.

### *DMDRMR* functions as a ceRNA for miR-378a-5p

**Fig. 3:**
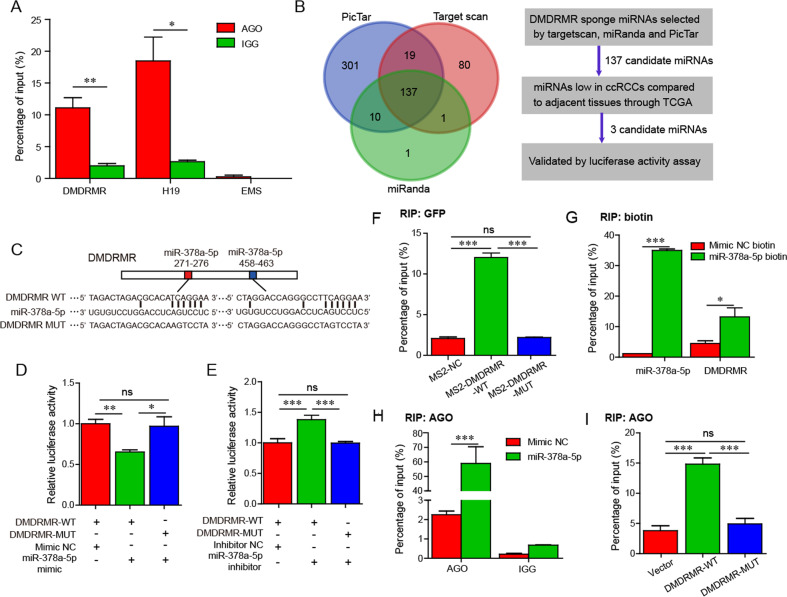
*DMDRMR* binds to miR-378a-5p. **A** Ago2 RIP qRT-PCR was performed to analyze the interaction of *DMDRMR* with Ago2 in 786-O cells. *H19* as a positive control and *EMS* as a negative control. **B** Flowchart showing stepwise approach to identify the potential *DMDRMR* bound to miRNAs. **C** Schematic representation shows two predicted binding sites for miR-378a-5p in *DMDRMR. DMDRMR* wild type (WT) sequence (upper) and mutant (MUT) sequence (lower). **D**, **E** Relative luciferase activities of *DMDRMR-*WT and MUT luciferase reporters in miR-378a-5p mimic (**D**) and inhibitor (**E**)-transfected 293T cells. **F** GFP RIP qRT-PCR assay showing the interaction of miR-378a-5p with *DMDRMR* in 293T cells. NC, negative control. **G** In vitro-synthesized biotin-labeled miR-378a-5p pull down assay showing the interaction of *DMDRMR* with miR-378a-5p in 786-O cells. **H**, **I** Ago2 RIP qRT-PCR assay showing the interaction of *DMDRMR* with Ago2 in miR-378a-5p mimic-transfected 786-O cells (**H**) and *DMDRMR-*WT and MUT OE 769-P cells (**I**). *p < 0.05, **p < 0.01, ***p < 0.001. ns, no significant.

We showed that *DMDRMR* partially localizes to the cytoplasm [7], suggesting a possible function as a competing endogenous RNA (ceRNA) to sequester miRNAs and regulate angiogenesis in ccRCC [9, 10]. The miRNA sponge is expected to form a complex with Argonaute2 (Ago2) [11], thus we first assessed whether *DMDRMR* forms a complex with Ago2. RIP qRT-PCR assays showed that *DMDRMR* was indeed enriched in the Ago2 immunoprecipitates, which was associated with lncRNA *H19* as a positive control [12] and *EMS* as a negative control [13] (Fig. 3A), suggesting that *DMDRMR* acts as a miRNA sponge. To identify potential target miRNAs of *DMDRMR*, we performed bioinformatic analysis by TargetScan, miRanda and PicTar databases [14–16] and found 137 miRNAs that have at least a site on *DMDRMR* (Fig. 3B), among which 3 miRNAs including miR-378a-5p, miR-532-5p and miR-199a-5p were significantly downregulated in TCGA ccRCC tissues versus adjacent tissues and further validated (Fig. S3A–C). Luciferase reporter assays (LRAs) showed miR-378a-5p mimic repressed and miR-378a-5p inhibitor enhanced the luciferase activity of full-length *DMDRMR* (*DMDRMR*-WT), which was not changed by miR-532-5p and miR-199a-5p (Fig. S3D, E), suggesting that *DMDRMR* might selectively sponge miR-378a-5p.

To further examine whether *DMDRMR* binds to miR-378a-5p, introduction of miR-378a-5p mimic or inhibitor selectively reduced or increased reporter activities of *DMDRMR*-WT, respectively, but not a construct with the two mutated miR-378a-5p binding sites (*DMDRMR-*MUT) (Fig. 3C–E). Further seeking evidence for this interaction, we performed MS2-based GFP RIP (Fig. S3F) and demonstrated that *DMDRMR*-WT was enriched for miR-378a-5p, but not *DMDRMR-*MUT (Fig. 3F). The specific association was further validated by affinity pull-down of endogenous *DMDRMR* using in vitro-synthesized biotin-labeled miR-378a-5p (Fig. 3G), collectively supporting the specific interaction between *DMDRMR* and miR-378a-5p. Furthermore, qRT-PCR assays showed that *DMDRMR* and miR-378a-5p inhibited each other’s expression (Fig. S3G, H). miRNAs are known to bind their targets and cause translational repression and/or RNA degradation in an Ago2-dependent manner [17]. To further determine whether *DMDRMR* was regulated by miR-378a-5p in such a manner, we performed RIP qRT-PCR and found that endogenous *DMDRMR* pull-down by Ago2 was largely enriched in miR-378a-5p-transfected cells (Fig. 3H). Also, endogenous miR-378a-5p pull-down by Ago2 was specifically enriched in *DMDRMR*-WT OE cells, but not in *DMDRMR*-MUT OE cells (Fig. 3I), indicating that miR-378a-5p bound to *DMDRMR* and then induced the degradation of *DMDRMR* in an Ago2-dependent manner. These results indicate that *DMDRMR* may function as a ceRNA for miR-378a-5p.

**Fig. 4:**
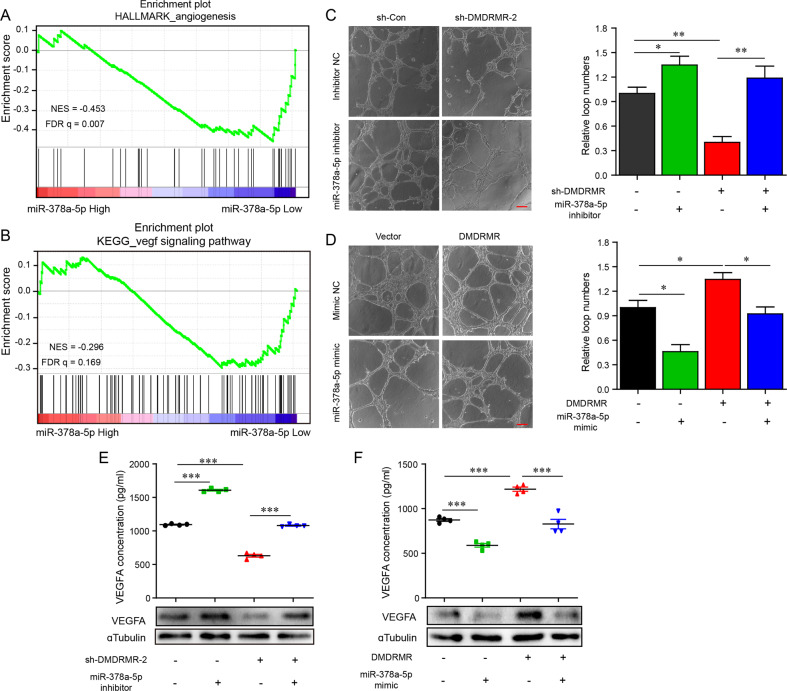
*DMDRMR* induces angiogenesis through sequestering miR-378a-5p. **A**, **B** GSEA showing the enrichment of “angiogenesis” (Hallmark database, **A**) and “vegf signaling pathway” (KEGG database, **B**) in high (red) and low (blue) miR-378a-5p expression. **C**, **D** Representative bright-field images (left) and quantifications (right) of matrigel tube formation of HUVECs incubated with conditioned medium from *DMDRMR* KD 786-O cells transfected with miR-378a-5p inhibitor (**C**) and *DMDRMR* OE 769-P cells transfected with miR-378a-5p mimic (**D**) (scale bars, 200 µm). **E**, **F** ELISA assay detecting the secreted VEGFA levels in *DMDRMR* KD 786-O cells transfected with miR-378a-5p inhibitor (**E**) and in *DMDRMR* OE 769-P cells transfected with miR-378a-5p mimic (**F**). The results are presented as mean ± SEM. *p < 0.05, **p < 0.01, ***p < 0.001. ns, no significant.

To elucidate whether miR-378a-5p mediates the promotional effect of *DMDRMR* on angiogenesis, we firstly performed GSEA analysis in TCGA ccRCC dataset and found that the gene signatures of angiogenesis and VEGF signaling pathways were enriched in patients with low miR-378a-5p expression (Figs. 4A, B and S4A–C), indicating that miR-378a-5p might inhibit angiogenesis and VEGF signaling pathways. Furthermore, rescue experiments showed that miR-378a-5p inhibitor reversed the inhibition of *DMDRMR* KD on the capillary tube formation of HUVECs, as well as protein and secreted levels of VEGFA (Fig. 4C, E). Conversely, miR-378a-5p mimic prevented the increased capillary tube formation of HUVECs and upregulated protein and secreted levels of VEGFA induced by *DMDRMR* OE (Fig. 4D, F). All these data suggest that an interaction with miR-378a-5p is necessary for *DMDRMR* to induce angiogenesis.

### *DMDRMR* increases EZH2 and SMURF1 expression through competitively binding miR-378a-5p

**Fig. 5:**
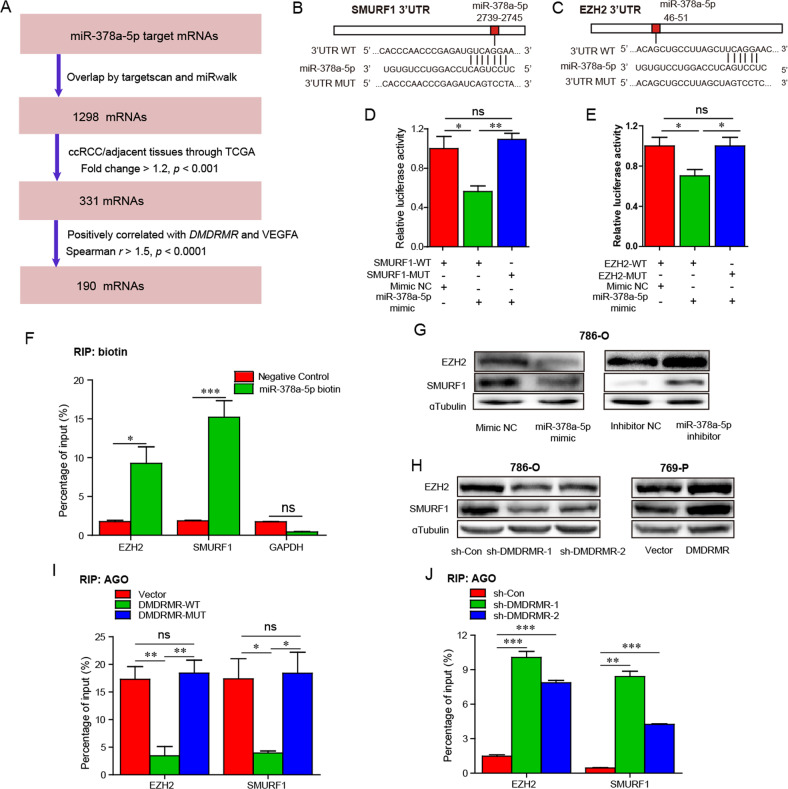
*DMDRMR* increases EZH2 and SMURF1 expression through sequestering miR-378a-5p. **A** Flow chart of identification of candidate mRNAs that miR-378a-5p targets. **B**, **C** Schematic representation shows the predicted binding sites for miR-378a-5p in the 3′UTR regions of SMURF1 (**B**) and EZH2 (**C**). The WT (upper) and MUT (lower) sequence of the 3′UTR regions of SMURF1 (**B**) and EZH2 (**C**). **D**, **E** Relative luciferase activities of SMURF1 (**D**) and EZH2 (**E**) 3′UTR-WT and MUT in miR-378a-5p mimic-transfected 293T cells. **F** In vitro-synthesized biotin-labeled miR-378a-5p pull down assay showing the interaction of EZH2 and SMURF1 with miR-378a-5p in 786-O cells. **G** Immunoblot of SMURF1 and EZH2 in miR-378a-5p mimic- (left) and inhibitor- (right) transfected 786-O cells. **H** Immunoblot of SMURF1 and EZH2 in *DMDRMR* KD 786-O cells (left) and *DMDRMR* OE 769-P cells (right). **I**, **J** Ago2 RIP qRT-PCR assay showing the interaction of EZH2 and SMURF1 with Ago2 in *DMDRMR-*WT and MUT OE 769-P cells (**I**) and in *DMDRMR* KD 786-O cells (**J**). The results are presented as mean ± SEM. *p < 0.05, **p < 0.01, and ***p < 0.001. ns, no significant.

To understand how *DMDRMR* promotes angiogenesis via miR-378a-5p, we used TargetScan [14] and miRwalk [18] to identify downstream targets of the miR-378a-5p, resulting in a set of 1298 target genes. 190 candidate target genes were selected according to the following criteria: upregulated in ccRCC tissues and positively correlated with both *DMDRMR* and *VEGFA* expression (Fig. 5A). By further considering well-studied oncogenes, we focused on SMAD specific E3 ubiquitin protein ligase 1 (SMURF1) and Enhancer of zeste homolog 2 (EZH2) that extensively regulate gene expression and thereby promote tumorigenesis in various carcinomas, including renal cancer [19, 20]. To explore whether miR-378a-5p could target *EZH2* and *SMURF1*, we performed LRAs and revealed that miR-378a-5p mimic repressed and miR-378a-5p inhibitor enhanced the luciferase activities of the 3′UTRs of both *EZH2* and *SMURF1*, but not 3′UTRs with mutations in miR-378a-5p targeting sites (Figs. 5B–E and S5A, B). Moreover, in vitro-synthesized biotin-labeled miR-378a-5p pulldown assays showed the direct binding of miR-378a-5p to the *EZH2* and *SMURF1* transcripts (Fig. 5F), suggesting that miR-378a-5p specifically targets the 3′UTR regions of *EZH2* and *SMURF1* transcripts. Furthermore, miR-378a-5p mimic decreased and miR-378a-5p inhibitor increased the expression levels of EZH2 and SMURF1 (Figs. 5G and S5C), confirming that miR-378a-5p inhibits expressions of *EZH2* and SMURF1 through directly targeting the two transcripts.

We further assessed whether *DMDRMR* is involved in the regulation of miR-378a-5p to EZH2 and SMURF1. Strikingly, *DMDRMR* KD downregulated, whereas its OE upregulated, EZH2 and SMURF1 expression (Fig. 5H). Furthermore, we performed RIP qRT-PCR assays and found that *DMDRMR*-WT OE, but not *DMDRMR*-MUT OE led to the reduced enrichment of Ago2 on EZH2 and SMURF1 transcripts (Fig. 5I). Conversely, *DMDRMR* KD elicited a significant increase in the recruitment of Ago2 to EZH2 and SMURF1 transcripts (Fig. 5J). The EZH2 small-molecule inhibitor is being evaluated in clinical trials for the treatment of cancers, including tazemetostat [21], thus we further evaluated the relevance of *DMDRMR*/miR-378a-5p axis in the efficacy of tazemetostat. Cell proliferation assay showed that miR-378a-5p mimic partially weakened the resistance of *DMDRMR* OE to tazemetostat (Fig. S5D), suggesting that miR-378a-5p could potentiate the efficacy of tazemetostat in ccRCC. These results demonstrate that *DMDRMR* increases EZH2 and SMURF1 expression by competitively binding miR-378a-5p.

### *DMDRMR* represses DAB2IP expression through its ceRNA

**Fig. 6:**
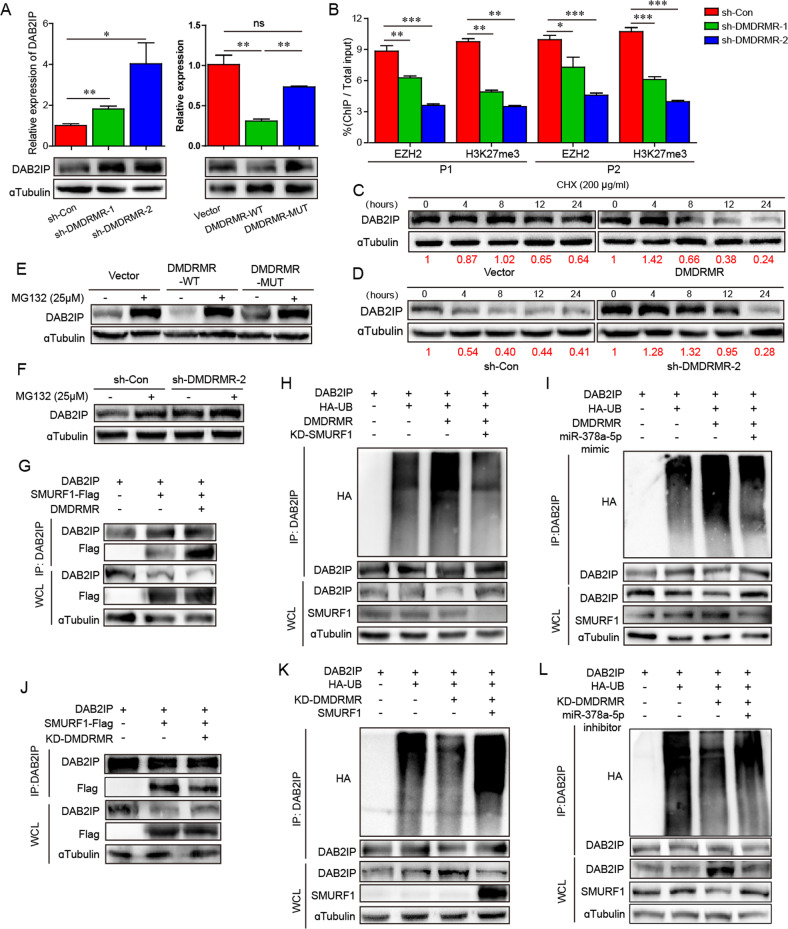
*DMDRMR* represses DAB2IP expression through its ceRNA. **A** The qRT-PCR (top) and immunoblot (bottom) analysis of DAB2IP in *DMDRMR* KD 786-O cells (left) and in *DMDRMR*-WT and MUT OE 769-P cells (right). **B** ChIP qRT-PCR analysis showing the binding efficiencies of EZH2 and H3K27me3 to the two regions of DAB2IP promoter in *DMDRMR* KD 786-O cells. P1, the first region of DAB2IP promoter; P2, the second region of DAB2IP promoter. **C**, **D** Immunoblot of DAB2IP in the *DMDRMR* OE 769-P cells (**C**) and KD 786-O (**D**) cells treated with cycloheximide (CHX) for indicated times. The densitometry analysis showing the relative DAB2IP expression levels that normalized to the reference protein αTubulin (Red numbers). **E**, **F** Immunoblot of DAB2IP in the *DMDRMR*-WT and MUT OE 769-P cells (**E**) and *DMDRMR* KD 786-O cells (**F**) treated with MG132 for 10 h. **G**, **J** Immunoprecipitation showing the association between DAB2IP and SMURF1 in *DMDRMR* OE 769-P cells (**G**) and KD 786-O cells (**J**). **H**, **K** Immunoprecipitation showing the associations between DAB2IP and HA-tagged ubiquitin (HA-UB) in SMURF1 KD with *DMDRMR* OE 293T cells (**H**) and in *DMDRMR* KD with SMURF1 OE 293T cells (**K**). **I**, **L** Immunoprecipitation showing the associations between DAB2IP and HA-UB in *DMDRMRM* OE transfected with miR-378a-5p mimic 293 T cells (**I**) and in *DMDRMR* KD transfected with miR-378a-5p inhibitor 293T cells (**L**). The results are presented as mean ± SEM. *p < 0.05, **p < 0.01, and ***p < 0.001.

DAB2IP, also known as a tumor suppressor in ccRCC, has been reported to be epigenetically repressed by EZH2-mediated methylation of lysine 27 in histone H3 (H3K27me3), and also be degraded by SMURF1-mediated ubiquitin-proteasome regulation [22–24]. Moreover, DAB2IP functions as an endogenous inhibitor in VEGFR2-mediated angiogenesis [25]. Thus, we hypothesized that *DMDRMR* antagonizes DAB2IP via its ceRNA. The qRT-PCR and immunoblot assays showed that both *DMDRMR* KD and KO increased, whereas *DMDRMR*-WT OE inhibited the transcriptional and protein levels of DAB2IP, which was not affected by *DMDRMR*-MUT OE (Figs. 6A and S6A, B). To further evaluate whether *DMDRMR* inhibits DAB2IP expression via EZH2 and SMURF1, we performed rescue experiments and showed that *DMDRMR* KD reversed the transcriptional level of DAB2IP depressed by EZH2 OE (Fig. S6C). Moreover, EZH2 OE or SMURF1 OE reduced DAB2IP protein level and abrogated the effects of *DMDRMR* silencing on DAB2IP expression (Fig. S6D, E). Similar effects were also observed in *DMDRMR* KD cells transfected with miR-378a-5p inhibitor (Fig. S6F). Conversely, EZH2 KD or SMURF1 KD promoted DAB2IP protein level and reversed the effects of *DMDRMR* OE on DAB2IP expression (Fig. S7A, B), which were also observed in *DMDRMR* OE cells transfected with miR-378a-5p mimic (Fig. S7C). Meanwhile, the increased and decreased expression levels of H3K27me3 were consistent with EZH2 (Figs. S6D–F and S7A–C). These results indicate that *DMDRMR* inhibits DAB2IP expression through EZH2 and SMURF1.

To further dissect underlying mechanisms of the decreased DAB2IP upon the increased *DMDRMR*, ChIP qRT-PCR and LRAs were first performed. Two putative sites of DAB2IP with potential promoter activities had been identified [26], thus we detected the effects of *DMDRMR* on the two sites of DAB2IP promoter. ChIP qRT-PCR assay revealed that *DMDRMR* KD reduced EZH2 and H3K27me3 occupancies on the two sites of DAB2IP promoter (Fig. 6B). Moreover, *DMDRMR*-WT OE but not *DMDRMR*-MUT OE inhibited, *DMDRMR* KD increased the luciferase activities of the two sites (Fig. S7D, E), suggesting that *DMDRMR* repressed the gene transcription of DAB2IP through facilitating the recruitment of EZH2 to DAB2IP promoter region accompanied with H3K27me3. Next, we examined whether *DMDRMR* mediates SMURF1-dependent DAB2IP degradation. We treated ccRCC cells with the protein synthesis inhibitor cycloheximide and assessed the protein levels of DAB2IP over time. Strikingly, *DMDRMR* OE decreased and *DMDRMR* KD increased the half-lives of DAB2IP protein (Fig. 6C, D). Following treatment with a proteasome inhibitor MG132, the inhibitory effects of *DMDRM*R-WT OE on DAB2IP protein level could be reversed (Fig. 6E). Conversely, the accumulation of endogenous DAB2IP in *DMDRMR* KD cells was increased (Fig. 6F), indicating that *DMDRMR* might accelerate the proteasome-dependent degradation of DAB2IP. Furthermore, *DMDRMR* OE enhanced the interaction between SMURF1 and DAB2IP (Fig. 6G), resulting in the increased ubiquitination level of DAB2IP, which was further attenuated by SMURF1 KD and miR-378a-5p mimic (Fig. 6H, I). In contrast, *DMDRMR* KD inhibited the association between SMURF1 and DAB2IP (Fig. 6J), and decreased DAB2IP ubiquitination that was rescued by SMURF1 OE and miR-378a-5p inhibitor (Fig. 6K, L), suggesting that *DMDRMR* attenuates the protein stability of DAB2IP through increasing the interaction of SMURF1 with DAB2IP and thereby promoting ubiquitination-dependent DAB2IP degradation. Collectively, these results indicate that *DMDRMR* represses DAB2IP expression through its ceRNA.

### DAB2IP is involved in *DMDRMR*-mediated angiogenesis

**Fig. 7:**
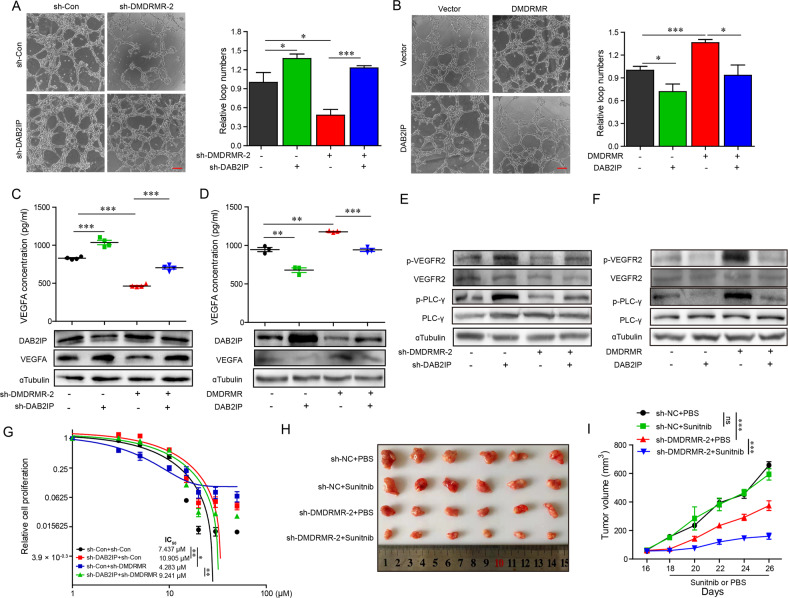
*DMDRMR* promotes angiogenesis through the decreased DAB2IP expression. **A**, **B** Representative bright-field images (left) and quantifications (right) of matrigel tube formation of HUVECs incubated with conditioned medium from *DMDRMR* KD 786-O cells with DAB2IP KD (**A**) and *DMDRMR* OE 769-P cells with DAB2IP OE (**B**) (scale bars, 200 µm). **C**, **D** ELISA assay detecting the secreted VEGFA levels in *DMDRMR* KD 786-O cells with DAB2IP KD (**C**) and in *DMDRMR* OE 769-P cells with DAB2IP OE (**D**). **E**, **F** Immunoblot of VEGFR2, p-VEGFR2, PLCγ and p-PLCγ of HUVECs incubated with conditioned medium from *DMDRMR* KD 786-O cells with DAB2IP KD (**E**) and *DMDRMR* OE 769-P cells with DAB2IP OE (**F**). **G** Cell proliferation assay assessing the half maximal inhibitory concentration (IC_50_) in 786-O cells transfected with indicated vectors following treatment with sunitinib for 5 days. **H**, **I** Subcutaneous xenograft assay of *DMDRMR* KD and control ACHN cells in nude mice with PBS or sunitinib treatment (**H**). Volumes of tumors are shown (*n* = 6 per group) (**I**). *p < 0.05, **p < 0.01, and ***p < 0.001. ns, no significant.

We next determined whether DAB2IP was functionally involved in *DMDRMR*-mediated angiogenesis. DAB2IP KD remarkably reversed the capillary tube formation of HUVECs and the protein and secreted levels of VEGFA which had been reduced by *DMDRMR* KD (Fig. 7A, C). Conversely, restoration of DAB2IP could impair the capillary tube formation of HUVECs and the protein and secreted levels of VEGFA induced by *DMDRMR* OE (Fig. 7B, D), supporting that DAB2IP is involved in *DMDRMR*-mediated angiogenesis.

DAB2IP is recruited to the VEGFR2-PLC-γ complex to inhibit VEGFR2-dependent angiogenic signaling [25], thus we explored whether *DMDRMR/*miR378a-5p/DAB2IP regulatory axis promotes angiogenesis through activating VEGFR2 signaling. We incubated HUVECs with conditional medium collected from ccRCC cells expressing variable amounts of *DMDRMR*, miR378a-5p or DAB2IP and performed immunoblot assays, the results showed that the inactive effects of *DMDRMR* KD on the phosphorylation of VEGFR2 and PLC-γ could be partially reversed by DAB2IP KD and miR-378a-5p inhibitor (Figs. 7E and S8A). Conversely, DAB2IP OE and miR-378a-5p mimic partially repressed the effect of *DMDRMR* OE on the phosphorylation of VEGFR2 and PLC-γ (Figs. 7F and S8B). These results demonstrated that *DMDRMR* activates VEGFA/VEGFR2 signaling through inhibiting DAB2IP expression, resulting in angiogenesis. Consequently, we further examined whether *DMDRMR* and DAB2IP regulate resistance of ccRCC cells to sunitinib, which targets angiogenic pathways. Cell proliferation assay showed that both *DMDRMR* KD and DAB2IP OE cells reduced sunitinib resistance (Fig. S8C, D). In contrast, *DMDRMR* OE and DAB2IP KD cells increased the resistance to sunitinib (Fig. S8C, D). Moreover, DAB2IP KD reversed the reduction of *DMDRMR* KD on the resistance to sunitinib (Fig. 7G). Similarly, *DMDRMR* OE also increased the other VEGFR inhibitor pazopanib resistance (Fig. S8E). Furthermore, in vivo xenograft experiments showed that *DMDRMR* KD increased ccRCC sensitivity to sunitinib as evidenced by the decreased volume and weight of tumors from mice treatment with sunitinib (Figs. 7H, I and S8F). These results suggest that *DMDRMR* enhances the resistance of ccRCC cells to sunitinib through DAB2IP.

### The ceRNA regulatory axis of *DMDRMR* is critical to ccRCC pathogenesis

**Fig. 8:**
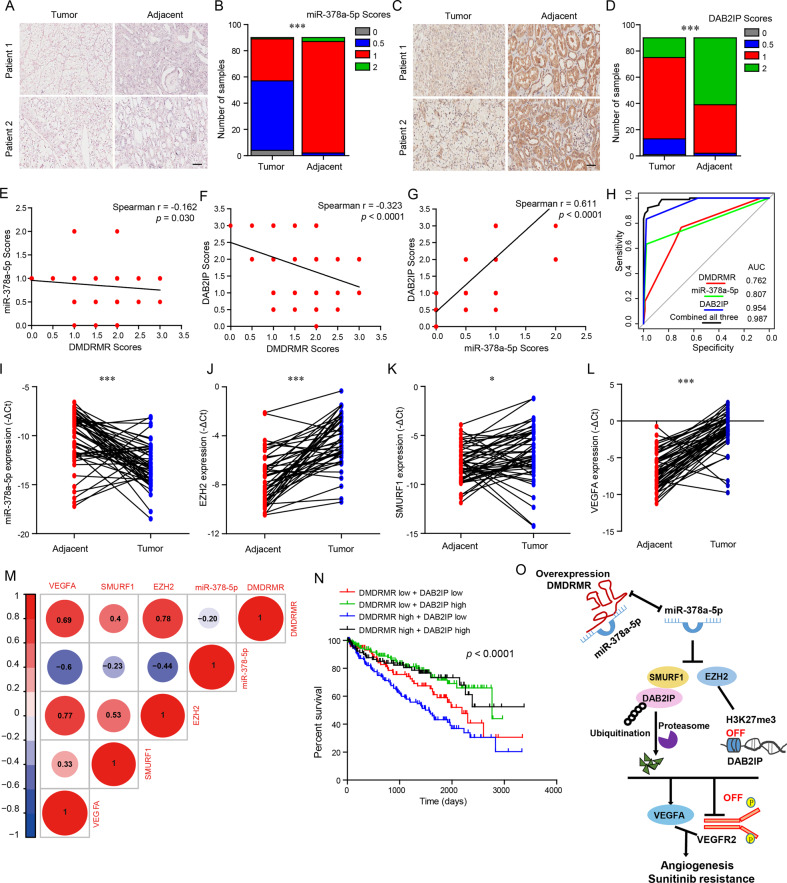
The correlation between *DMDRMR* and its ceRNA axis in ccRCC. **A**, **B** Representative images (**A**) and scores (**B**) of miR-378a-5p expression from ccRCC and adjacent tissues by ISH assays (scale bars, 50 µm). **C**, **D** Representative images (**C**) and scores (**D**) of DAB2IP expression from ccRCC and adjacent tissues by IHC assays (scale bars, 50 µm). **E**–**G** Spearman correlation analysis between miR-378a-5p and *DMDRMR* expression levels (**E**), DAB2IP and *DMDRMR* expression levels (**F**), DAB2IP and miR-378a-5p expression levels (**G)**. **H** ROC curve analysis for the indicated parameters in ccRCC compared to adjacent tissues using the expression levels of *DMDRMR*, miR-378a-5p, DAB2IP, and combined their expression levels. **I**–**L** The qRT-PCR analysis of miR-378a-5p (**I**), *EZH2* (**J**), *SMURF1* (**K**), and *VEGFA* (**L**) expression levels in 48 paired ccRCC and adjacent tissues. Delta cycle threshold (ΔCt). **M** The correlation analysis of *DMDRMR*, miR-378a-5p, *EZH2*, *SMURF1* and *VEGFA* expression levels. **N** Kaplan–Meier survival analysis was compared among the different combination of *DMDRMR* and *DAB2IP* expression. **O** Proposed model for the *DMDRMR/*miR-378a-5p/DAB2IP axis promoting the angiogenesis of ccRCC. The results are presented as mean ± SD. *p < 0.05 and ***p < 0.001.

To investigate the clinical relevance of the above findings, the same tissue microarray with our previous study [7] was assessed to evaluate the relationships among *DMDRMR*, miR-378a-5p, and DAB2IP. miRNA in situ hybridization (ISH) result revealed that, miR-378a-5p expression in the cytoplasm reduced in the ccRCC tissues versus the adjacent tissues (Fig. 8A, B). Similar result was observed in DAB2IP immunohistochemistry (IHC) staining (Fig. 8C, D). *DMDRMR* level was inversely correlated with miR-378a-5p and DAB2IP expression (Fig. 8E, F). Meanwhile, miR-378a-5p expression was positively correlated with DAB2IP expression (Fig. 8G). Furthermore, receiver operating characteristic (ROC) analysis showed that combined use of *DMDRMR*, miR-378a-5p and DAB2IP expression could improve the area under curve (AUC) value for the diagnosis of ccRCC (Fig. 8H), suggesting that the combination of *DMDRMR*, miR-378a-5p and DAB2IP expression can serve as a diagnostic biomarker of ccRCC.

Next, qRT-PCR assay was used to determine the association between *DMDRMR* and its ceRNA in 48 pairs of ccRCC tissues and adjacent tissues used in our previous study [7]. Downregulated miR-378a-5p levels and upregulated *EZH2*, *SMURF1*, and *VEGFA* transcriptional levels were observed in ccRCC tissues versus adjacent tissues (Fig. 8I–L). Moreover, spearman correlation analysis showed that *DMDRMR* was positively correlated with *EZH2*, *SMURF1*, and *VEGFA*, whereas miR-378a-5p was negatively correlated with *DMDRMR*, *EZH2*, *SMURF1*, and *VEGFA* (Fig. 8M). However, DAB2IP transcriptional level was increased in ccRCC tissues (Fig. S9A). Furthermore, we also analyzed the TCGA ccRCC dataset, high expression levels of *EZH2* and *SMURF1* and low expression of *DAB2IP* predicted poor overall survival of ccRCC patients (Fig. S9B–D). High *EZH2* and low *DAB2IP* expression was associated with poor outcomes, including pathologic stage, tumor size, metastatic status and Fuhrman grade (Fig. S9B, D). These results supported the oncogenic role of EZH2, SMURF1, and VEGFA and the tumor suppressive role of miR-378a-5p and DAB2IP in ccRCC. Consistent with our findings, DAB2IP was negatively correlated with *DMDRMR* and EZH2 (Fig. S9E, F). Interestingly, the combination of high *DMDRMR* and low DAB2IP predicted the poorest overall survival of patients (Fig. 8N). These results demonstrated that the ceRNA regulatory axis of *DMDRMR* is clinically relevant to ccRCC pathogenesis and prognosis of patients with ccRCC.

## Discussion

Angiogenesis is a hallmark of cancer, which supplies enough oxygen and nutrients to promote the progression of ccRCC [27]. Consistent with our study, several other studies had reported that lncRNAs promote tumor angiogenesis or sunitinib resistance in RCC, including *HOTAIR* and *lncARSR* [28, 29], supporting that lncRNAs may provide new targets for therapy and predictive biomarkers for anti-angiogenesis therapy response in RCC. It is established that lncRNAs function as ceRNA to increase VEGFA expression by competing with miRNAs, resulting in tumor angiogenesis [30–32]. However, our results revealed that VEGFA is an indirect-regulated gene of *DMDRMR*/miR-378a-5p axis and contribute to the effect of this axis on angiogenesis in ccRCC. Similar findings have been reported that miR-378a-5p can suppress angiogenesis of oral squamous cell carcinoma by targeting KLK4 and indirectly reducing VEGFA expression [33] or promotes tumor angiogenesis by directly and indirectly upregulating VEGFA, including metastatic melanoma [34, 35], which may be explained by the cancer-type specificity, cellular-context dependence, or selection of signaling pathway. Recent study demonstrated that miR-378a-5p can attenuate cell proliferation, migration, invasion and promote cell apoptosis in RCC, and is associated with good prognosis of patients with RCC [36], which further verify our finding that miR-378a-5p acts as a tumor suppressor in ccRCC. It has been reported that both EZH2 and SMURF1 exert oncogenic functions in ccRCC [19, 20, 37]. Loss of DAB2IP could enhance tumor growth and resistance to mTOR-targeted therapies and ionizing radiation in RCC [22]. However, the dysregulated mechanism of EZH2, SMURF1 and DAB2IP expression remains enigmatic. Herein, we discovered that *DMDRMR*/miR-378a-5p axis decreases DAB2IP expression through directly upregulating EZH2 and SMURF1 in ccRCC. Moreover, we extend the role of miR-378a-5p, EZH2, SMURF1, and DAB2IP in ccRCC angiogenesis.

DAB2IP functions as endogenous inhibitor of adaptive angiogenesis in part by binding directly to VEGFR2 and limiting PI3K activation [25]. Although we did not define how the *DMDRMR*/miR-378a-5p/DAB2IP axis regulates VEGFA expression, we revealed that *DMDRMR*/miR-378a-5p axis inhibits the inactivation of DAB2IP-regualted VEGFA/VEGFR2 signaling pathway, which can explain our notion that the *DMDRMR*/miR-378a-5p axis could serve as an angiogenic activator in ccRCC. Given the known function of *DMDRMR* and miR-378a-5p in ccRCC [7, 36], and angiogenesis has been shown to be responsible for tumor growth and metastasis [38], we also explored whether the *DMDRMR/*miR-378a-5p/DAB2IP axis regulates the cell proliferation, migration, invasion of ccRCC and found that *DMDRMR* KD partially abrogated the stimulative effect of miR-378a-5p inhibitor and DAB2IP KD on these phenotypes (Fig. S10A–F), suggesting that *DMDRMR* promotes the cell proliferation, migration, invasion of ccRCC through selectively repressing miR-378a-5p and DAB2IP. Therefore, this study further reinforces our previous finding that *DMDRMR* promotes tumor growth and metastasis [7]. Clearly, this study will improve our understanding of the mechanistic, functional, and pathological roles of *DMDRMR* in ccRCC. But whether the function and molecular mechanism of *DMDRMR* is involved in other cancers need to be further investigated.

Exosomal lncRNAs had been identified as promising biomarkers for cancers [39]. ExoRBase, a repository of extracellular vehicles (EVs) lncRNAs derived from blood of human healthy and cancer cohorts [40], showed that *DMDRMR* was expressed in EVs and higher expression levels in the esophageal squamous cell carcinoma, gastric cancer and melanoma relative to healthy cohorts (Fig. S11). However, EVs *DMDRMR* was not statistically significant in ccRCC cohort, which may be explained by small sample size. Targeted nucleic acid-based therapeutics are emerging as a promising approach [41, 42], therefore, we need to further explore the clinical value of oncogenic *DMDRMR* in exosomal biomarkers and nucleic acid therapeutic targets for cancers in future.

Overall, our study reveals that *DMDRMR* is a pro-angiogenic lncRNA that promotes angiogenesis and sunitinib resistance, by competitively binding miR-378a-5p to promote EZH2 and SMURF1-mediated repression of DAB2IP expression and thereby activating VEGFA/VEGFR2 signaling pathway (Fig. 8O). This pathway might provide novel clinical markers and therapeutical targets for ccRCC patients.

### Reporting Summary

Further information on research design is available in the Nature Research Reporting Summary linked to this article.

## Supplementary information

Supplementary file

Original western blots

Reporting Summary

## Data Availability

The data and materials during this study are available from the corresponding author on reasonable request.
