# Supplementary material for: DMDRMR promotes angiogenesis via antagonizing DAB2IP in clear cell renal cell carcinoma
Source: Cell Death Dis. 2022 May 13;13(5):456. doi: 10.1038/s41419-022-04898-3 (PMC9106801; doi:10.1038/s41419-022-04898-3)
Supplement: Supplementary file 1 — Supplementary file [file 41419_2022_4898_MOESM1_ESM.docx]

***DMDRMR* Promotes Angiogenesis via Antagonizing DAB2IP in clear cell Renal Cell Carcinoma**

Yumeng Zhu^a,b,1^, Xiaojun Liu^a,b,1^, Yang Wang^c^,Yongbo Pan^c^, Xiaoqi Han^d^, Bo Peng^a,b^, Xu Zhang^e^, Shaoxi Niu^e^, He Wang^b^, Qinong Ye^f^, Yinmin Gu^b,g,2^ and Shan Gao^b,c,h,2^

Corresponding author: Shan Gao; Yinmin Gu

**Supplementary Materials and Methods**

**Quantitative real time reverse transcription polymerase chain reaction (qRT-PCR)**

Total RNA was prepared using RNAiso Plus (Takara, 9109) according to the manufacturer’s instructions. PrimeScript RT reagent kit with gDNA eraser (Takara, RR047A) was used to synthesize the first-strand cDNA from total RNA. qRT-PCR was performed using TB Green Premix Ex Taq (Takara, RR820A) with a QuantStudio7 Flex System. Each sample was run in triplicate, and the relative gene expression levels were normalized against an internal control (GAPDH) and calculated using the 2^-ΔΔCT^ method (1).

**Immunoblot**

Cells were washed with cold phosphate buffer saline (PBS) and lysed using radio-immunoprecipitation assay (RIPA) lysis buffer (Beyotime,China) containing 1% protease and phosphatase inhibitor cocktails (Sigma, Aldrich, Burlington, MA, USA)) for 30 min on ice, centrifuged at 12,000 rpm for 20 min at 4°C. The proteins in the supernatant were collected and measured by using bicinchoninic acid (BCA) assay kit (Beyotime, China). Protein samples were boiled for 10 min, resolved in 10% sodium dodecyl sulfate (SDS)-polyacrylamide gels and transferred to polyvinylidene difluoride (PVDF) membranes (Millipore, Burlington, MA, USA). After being blocked with 5% nonfat milk diluted in trisphosphate buffered saline (TBS) containing 0.05 % Tween-20 (TBST) for 1 hour (h), the membranes were incubated with the specific primary antibodies at 4 °C overnight. The membranes were then washed three times with TBST and incubated with corresponding horseradish peroxidase (HRP)-conjugated secondary antibody at a 1:2000 dilution for 2 h at room temperature (RT). The signals were then visualized using the immobilon western horseradish peroxidase substrate kit (Millipore).

**Immunohistochemistry (IHC)**

The tumor tissues of mouse were fixed with 4% paraformaldehyde. The sections were paraffin embedded, sectioned in 5 μm, dewaxed, hydrated, and washed. After neutralization of endogenous peroxidases, slides were pre-incubated with blocking serum and then incubated at 4 °C overnight with primary antibody. Subsequently, sections were serially rinsed, incubated with tested for CD31 and VEGFA protein expression with the diaminobezidin (DAB) IHC Detection kit (Origene). Images were taken using an OPLENIC digital camera mounted on a light microscope (40×objective) and quantified using the Image J software.

**miRNA locked-nucleic acid (LNA) *in situ* hybridization (ISH)**

Hsa-miR-378a-5p detection probe (3’and 5’- end labelled with digoxigenin (DIG) and LNA-modified) was purchased from Exiqon. In a tissue array that was purchased from the Shanghai Outdo Biotech Co.,Itd (Shanghai, China), expression of miR-378a-5p was examined within ISH. Briefly, the tissue array was deparaffinized in fresh xylene, subjected to dehydrated gradient dehydration in ethanol solution and air dried. Then, 20 μg/ml of proteinase K was applied onto the tissue array and placed in the hybridization chamber at 37 °C for 30 min. The DIG-labeled probe was denatured at 90 °C for 4 min and hybridized in the tissue array at 50 °C for 15 h. After washing, tissues were incubated overnight at 4°C with anti-DIG-Alkaline Phosphatase (AP) antibody (Boster) and then were dyed using AP Assay Kit (Beyotime). Cell nuclei counterstained with 0.1% Nuclear Fast Red solution (Solarbio, Beijing, China).

**Nuclear run on assay**

Nuclear run on assay was performed as previously described (2). Briefly, the nuclei were isolated from *DMDRMR* knockdown (KD) or knockout (KO) cells, followed by *in vitro* transcription in the presence of biotinylated-16-UTP, and purification of RNA transcripts using Dynabeads M-280 Streptavidin. Finally, the purified RNAs were reversed to cDNAs and then subjected to qRT-PCR to measure the transcription efficiency of VEGFA. GAPDH acts as an internal control.

**RNA immunoprecipitation (RIP) assay**

The RIP assay was performed using the EZ-Magna RIP™ RNA-Binding Protein Immunoprecipitation Kit (Merck Millipore, 17-700) according to the manufacturer’s instructions. Briefly, approximately 2×10^7^ cells were lysed with 200 μl RIP lysis buffer supplemented with 1 μl of protease inhibitor cocktail and 0.5 μl of RNAase inhibitor, then incubated with antibody-coated A/G magnetic beads at 4 °C overnight. After washing using wash buffer for six times, the bounded immunocomplexes were re-suspended in 150 µl of proteinase K buffer (117 µl of RIP Wash Buffer, 15 µl of 10% SDS, and 18 µl of proteinase K) and incubated at 55°C for 30 min with constant shaking. The coprecipitated RNA was purified using RNeasy® Mini Kit (Qiagen, 74106), subjected to cDNA synthesis and then for qRT-PCR analysis.

**Chromatin immunoprecipitation (ChIP) assay**

ChIP was performed with Magna ChIP HiSens Chromatin IP Kit (Merck Millipore, 17-10460) according to the manufacturer's instructions. In brief, cells were washed with PBS for 3 times and centrifuged at 500×g for 10min. Then cells were cross-linked with 4% formaldehyde for 10 min at 37°C, and then sonicated using a covaris M220 to generate 200-500 bps DNA fragments. Samples were incubated with anti-EZH2 or H3K27me3 antibody on a rotator at 4 °C overnight. DNA was purified and then subjected to qRT-PCR.

**Dual luciferase reporter gene assay**

*DMDRMR* full length (*DMDRMR*-WT), *DMDRMR*-containing the mutant predictive binding sites (BSs) of miR-378a-5p (*DMDRMR*-MUT), EZH2/SMURF1 3’UTR (EZH2/SMURF1-WT) and EZH2/SMURF1 3’UTR-containing the mutant predictive BSs of miR-378a-5p (EZH2/SMURF1-MUT) were cloned into pmirGLO Dual-Luciferase miRNA Target Expression vector (Promega, E133A). These plasmids were individually co-transfected with miR-378a-5p mimic or inhibitor into 293T cells using JetPRIME ® DNA and siRNA Transfection Reagent (Polyplus, 114-15). Similarly, two putative promoter regions (P1, fragment spanning from +229 to +981; P2, fragment spanning from -598 to +44 relative to the transcription start site of the DAB2IP) were cloned into pmirGLO Dual-Luciferase miRNA Target Expression vector. *DMDRMR* OE/*DMDRMR* KD vectors were co-transfected with pmirGLO-DAB2IP-P1 or pmirGLO-DAB2IP-P2 into 293T cells. Each group was run in a 24-well plate. The luciferase activities were detected by Dual-Luciferase Reporter Assay System (Promega, E292) after 48 h of transfection according to the manufacturer’s instructions. Renilla luciferase activity was normalized against firefly luciferase activity.

**Half maximal inhibitory concentration (IC_50_)**

The indicated cells were seeded into 96-well plates at a density of 5×10^3^cells/well overnight. Different concentration of sunitinib was added to each well. After 5 days, cell viability was measured using CellTiter-Glo Luminescent™ Cell Viability Assay kit (Promega, G7573) and a Bio-Rad microplate reader. IC_50_ values were calculated by probit analysis in calculated using GraphPad Prism 8.0 (GraphPad software, Inc.).

**Coimmunoprecipitation (Co-IP)**

Cells were lysed with Cell lysis buffer for Western and IP (Beyotime) with protease inhibitor cocktail (Sigma, P8340) on ice, and DAB2IP antibody was incubated overnight at 4°C with 1mg of protein samples, and followed by incubating with PureProteome™ Protein A/G Mix Magnetic Beads (Millipore, LSKMAGAG02). The-bounded immunocomplexes were washed six times with lysis buffer, suspended in 5×loading buffer and lysis buffer, and boiled for 15 min, and then followed by immunoblot assay.

**Migration and invasion assay**

Invasion assays were performed in Transwell chamber: 24-well, 8.0-μm pore membranes (Corning). 2×10^4^ cells in serum free media were plated in the upper chamber (Corning), while 600 μl medium supplemented with 10% FBS were added into the lower chamber and served as a chemotactic agent, and incubated for 24 h to allow the cells to migrate to the lower chamber. After incubation, cells that migrated through the filters were fixed with 95% ethanol and stained with crystal violet. The stained cells were destained with 33% acetic acid. The ability of cell invasion or migration was then assessed by measuring the absorbance at 560 nm. The migration assay was conducted similarly, without coating the filters with Matrigel.

**Tumor xenograft model**

Five-week-old male BALB/c nude male mice were chosen for the tumor xenograft model. A total of 5 × 10^6^ *DMDRMR* KD and control ACHN cells subcutaneously injected into the flanks of each mouse (n = 12 mice/group). Tumor sizes were measured every 2 or 3 days. After 18 days, mice with size-matched tumors were randomized into two treatment groups. 6 mice in the *DMDRMR* KD group and 6 mice in the control group received intraperitoneal injection of 40 mg/kg/day of sunitinib in PBS/1% DMSO or vehicle injections (PBS/1%DMSO). Mice were killed after 26 days. Tumor sizes and wights were measured. All protocols involving animals were previously approved by the Ethics Committee for the Use of Experimental Animals of the Suzhou Institute of Biomedical Engineering and Technology, Chinese Academy of Sciences (Suzhou, Jiangsu, China).

**Sequences and antibodies**

Sequences of short hairpin RNA (shRNA) for KD and primer sequences and antibodies used are listed in Supplementary Table S1-S4.

**Supplementary Figures
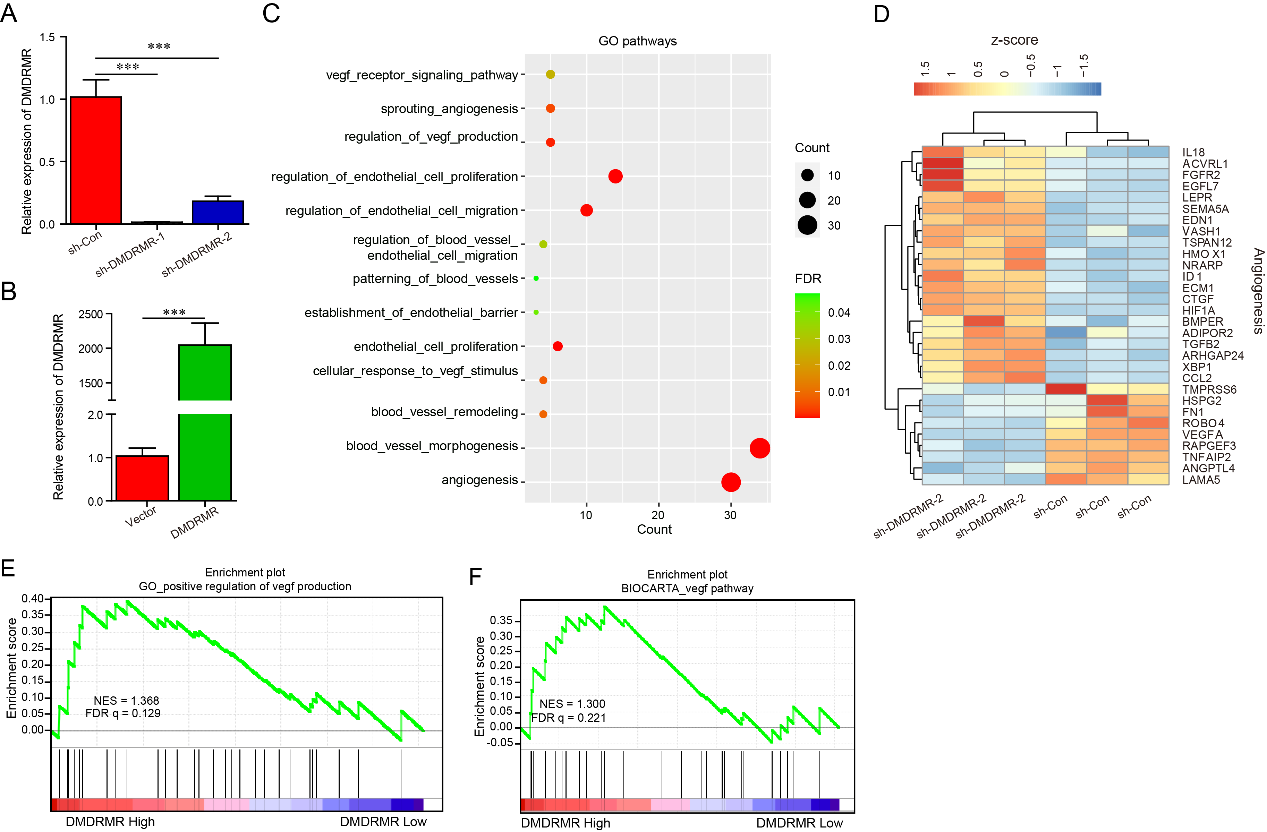
**

**Figure S1. *DMDRMR* involves in angiogenesis-related pathways.** (A-B) The qRT-PCR analysis of *DMDRMR* in *DMDRMR* KD 786-O cells (A) and OE 769-P (B) cells. (C) The 13 GO pathways related to angiogenesis are identified by the differentially expressed genes (DEGs) of *DMDRMR* KD and control 786-O cells. The color intensities represent the false discovery rate (*FDR*). The circle sizes represent the number of DEGs. (D) Heat map showing the angiogenesis-related genes regulated by *DMDRMR*. (E-F) GSEA data showing the enrichment of “positive regulation of vegf production” (GO terms, E) and “vegf pathway” (Biocarta database, F) in high (red) and low (blue) *DMDRMR* expression.

**
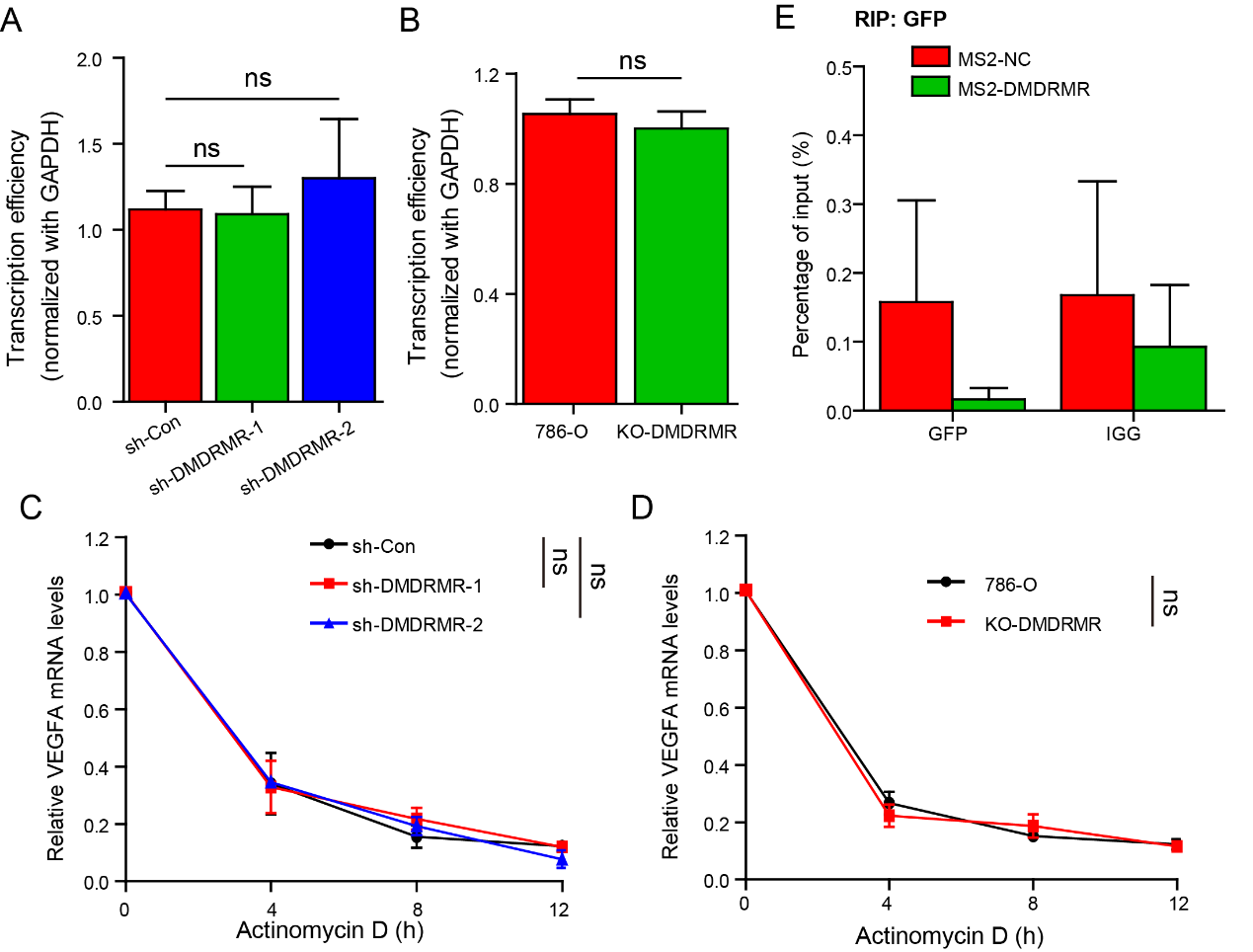
Figure S2. *DMDRMR* indirectly regulates VEGFA.** (A-B) The nuclear run-on assay showing the transcriptional efficiencies of *VEGFA* in *DMDRMR* KD (A) and KO (B) 786-O cells. (C-D) The half-lives of *VEGFA* mRNA in the *DMDRMR* KD (C) and KO (D) 786-O cells after treatment with 5 μM actinomycin D for the indicated times. (E) GFP RIP qRT-PCR assay showing the interaction of the *VEGFA* with *DMDRMR* in 293T cells. IgG as a negative control. Results are presented as the mean ± SEM. ns, not significant.

**
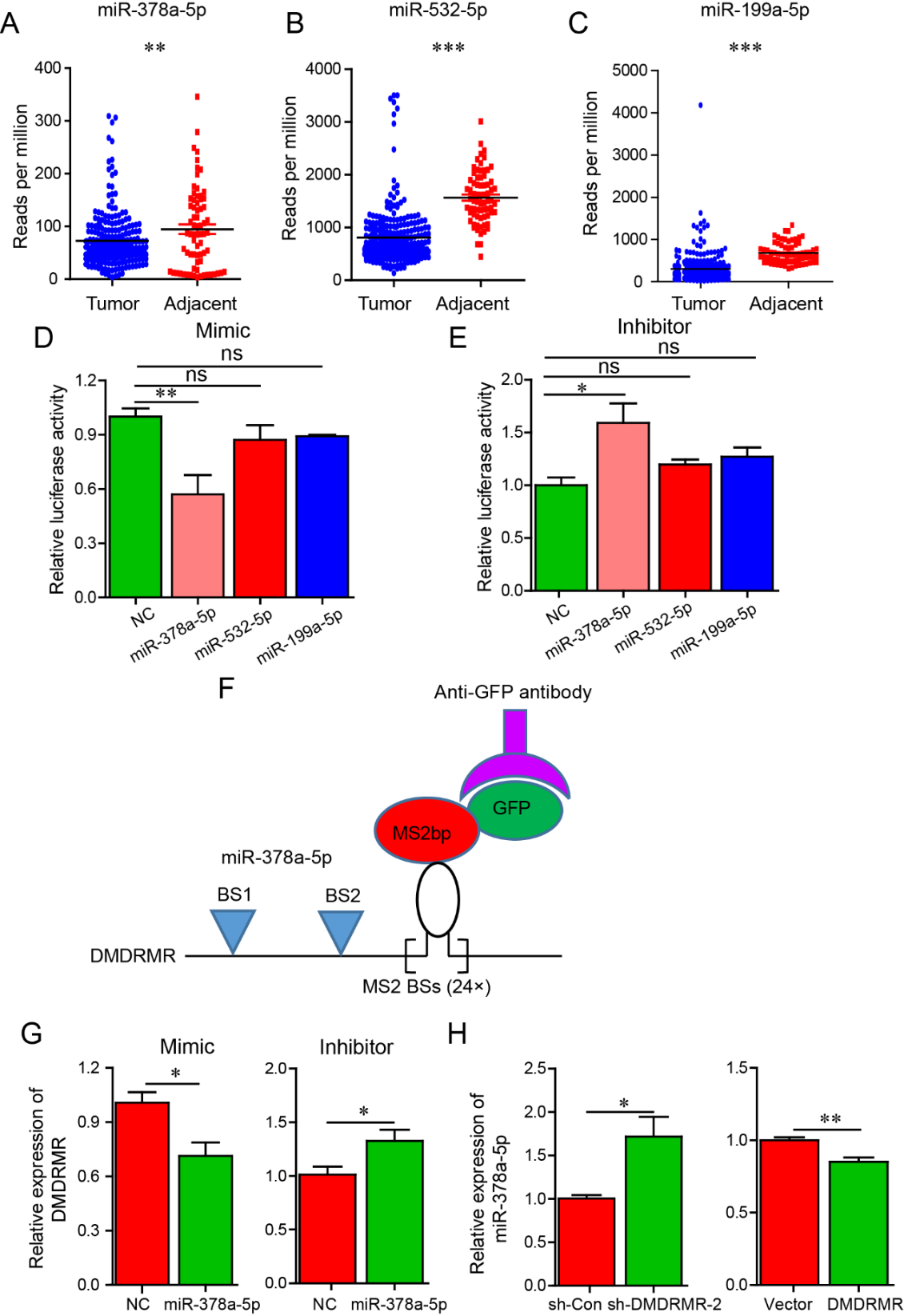
**

**Figure S3. *DMDRMR* and miR-378a-5p inhibit each other’s expression.** (A-C) The expression levels of miR-378a-5p (A), miR-532-5p (B) and miR-199a-5p (C) in tumors and adjacent tissues from TCGA ccRCC cohort. (D-E) Relative luciferase activities of *DMDRMR-*WT reporter in 293T cells transfected with miR-378a-5p, miR-532-5p or miR-199a-5p mimic (D) and each inhibitor (E). (F) Schematic illustration for experimental design of GFP-RIP followed by qRT-PCR assay to detect miR-378a-5p endogenously associated with *DMDRMR*. (G) The qRT-PCR analysis showing the expression levels of *DMDRMR* in miR-378a-5p mimic-transfected 769-P cells (left) and inhibitor-transfected 786-O cells (right). (H) The qRT-PCR analysis showing the expression levels of miR-378a-5p in *DMDRMR* KD 786-O (left) and OE 769-P (right) cells. Results are presented as the mean ± SEM. **p* < 0.05, ***p* <0.01, and ****p* < 0.001.

**
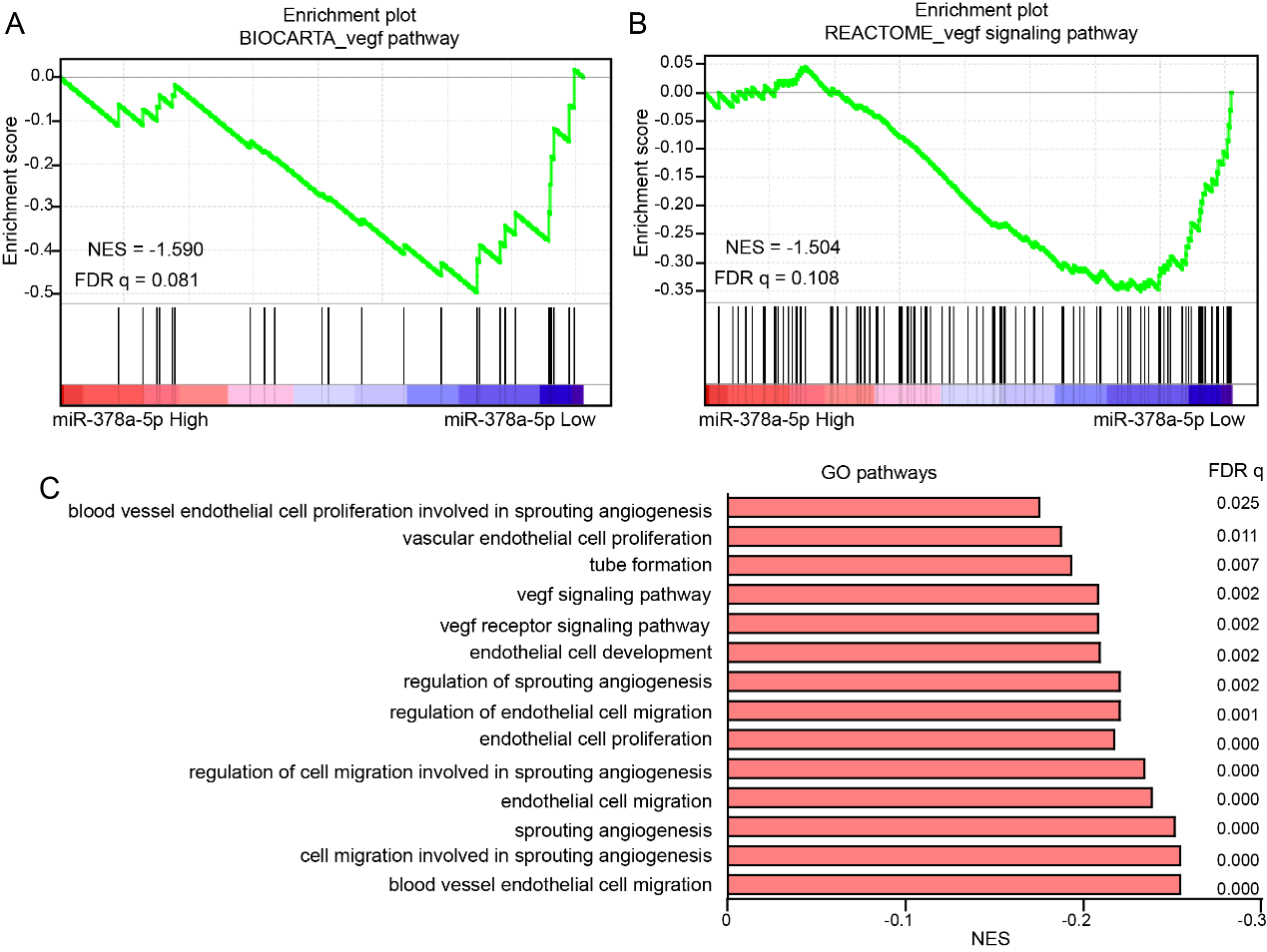
Figure S4. miR-378a-5p involves in angiogenesis-related pathways.** (A-B) GSEA data showing the enrichment of “vegf pathway” (Biocarta database, A) and “vegf signaling pathway” (Reactome database, B) in high (red) and low (blue) miR-378a-5p expression. (C) GSEA data showing the enrichment of angiogenesis-related pathways in high and low miR-378a-5p expression using GO terms.

**
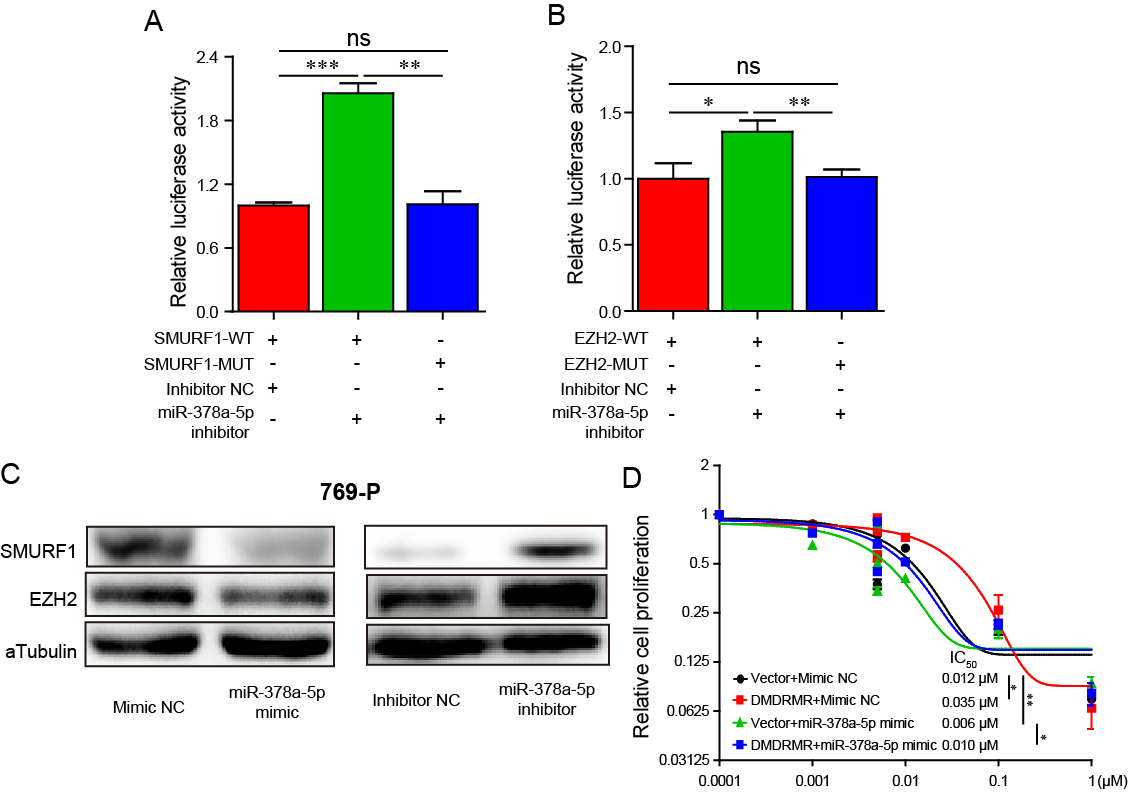
**

**Figure S5. miR-378a-5p inhibits the expression of EZH2 and SMURF1.** (A-B) Relative luciferase activities of SMURF1 (A) and EZH2 (B) 3’UTR-WT and MUT in miR-378a-5p inhibitor-transfected 293T cells. (C) Immunoblot of SMURF1 and EZH2 in miR-378a-5p mimic- (left) and inhibitor- (right) transfected 769-P cells. (D) Cell proliferation assay assessing the half maximal inhibitory concentration (IC50) in 786-O cells transfected with indicated vectors following treatment with tazemetostat for 3 days. The results are presented as mean ± SEM. **p* < 0.05, ***p* < 0.01, and ****p* < 0.001. ns, not significant.

**
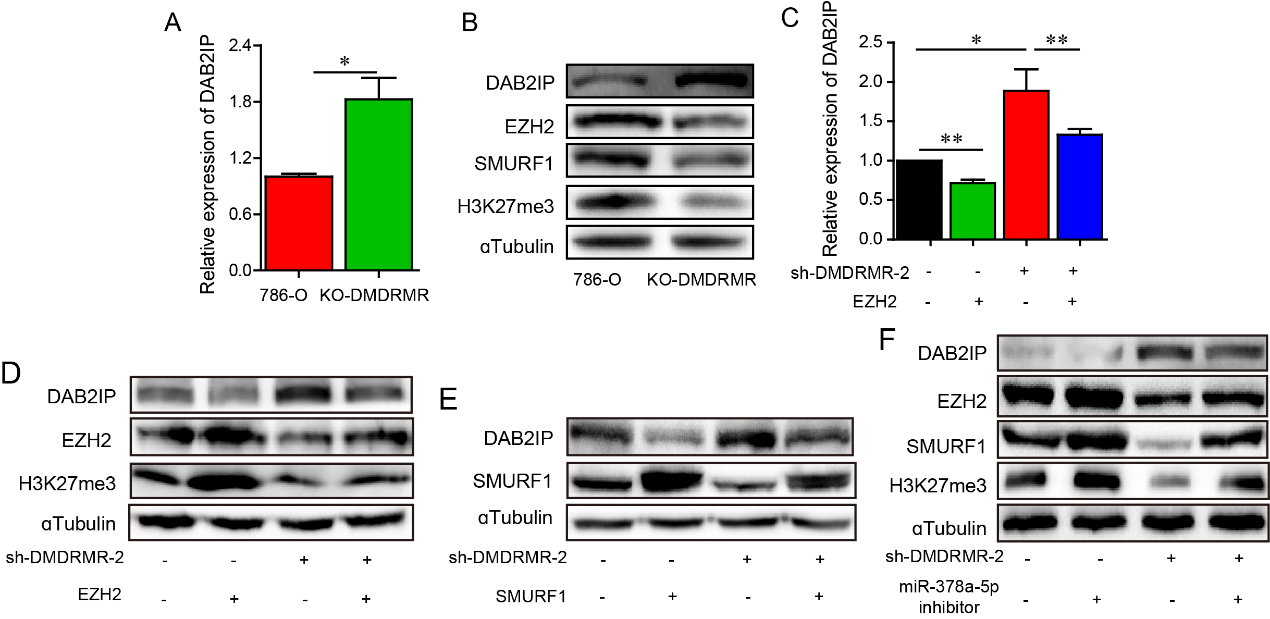
Figure S6. miR-378a-5p inhibitor represses the elevation of *DMDRMR* KD on DAB2IP expression.** (A) The qRT-PCR analysis of DAB2IP expression level in *DMDRMR* KO 786-O cells. (B) Immunoblot of DAB2IP, EZH2, SMURF1 and H3K27me3 in *DMDRMR* KO 786-O cells. (C) The qRT-PCR analysis of *DAB2IP* expression levels in *DMDRMR* KD 786-O cells with EZH2 OE. (D-F) Immunoblot of the indicated proteins in *DMDRMR* KD 786-O cells with EZH2 OE (D) or SMURF1 OE (E) or miR-378a-5p inhibitor (F). The results are presented as mean ± SEM. **p* < 0.05, ***p* < 0.01.


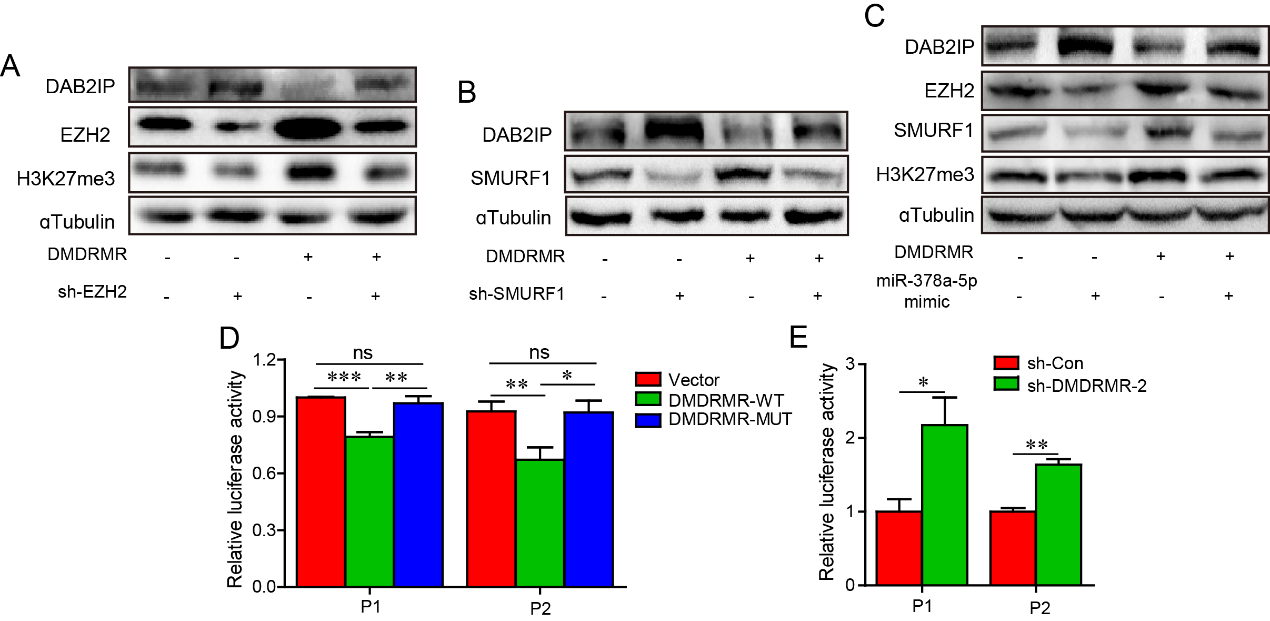


**Figure S7. miR-378a-5p mimic reverses the reduction of *DMDRMR* OE on DAB2IP expression.** (A-B) Immunoblot of the indicated proteins in *DMDRMR* OE 769-P cells with EZH2 KD (A) or SMURF1 KD (B) or miR-378a-5p mimic (C). (D-E) Relative luciferase activities of the two regions of DAB2IP promoter in *DMDRMR*-WT and MUT OE 293T cells (D) and in *DMDRMR* KD 293T (E) cells. The results are presented as mean ± SEM. * *p* < 0.05, ** *p* < 0.01, and ****p* < 0.001. ns, not significant.

**
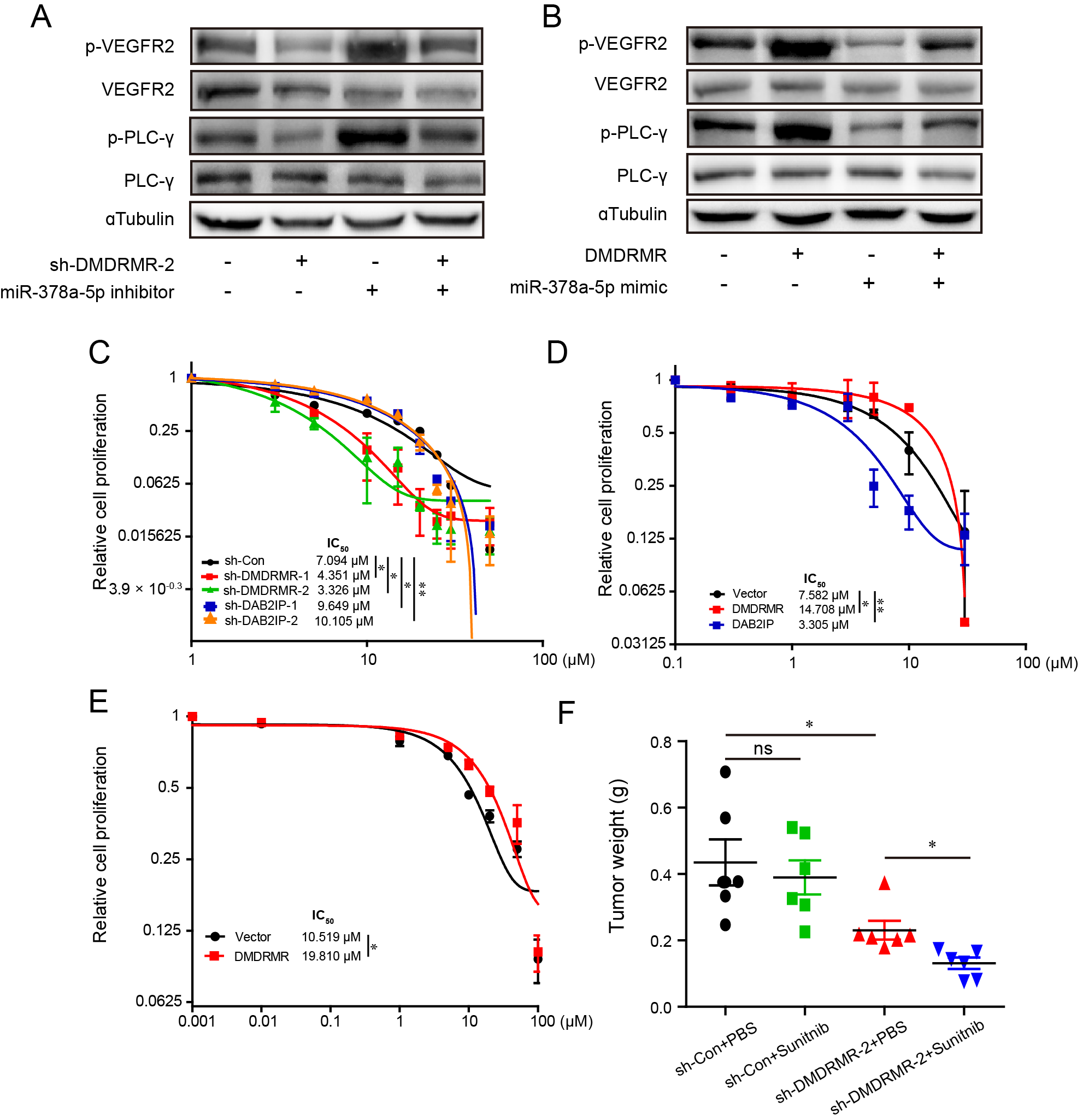
**

**Figure S8. *DMDRMR*/miR378a-5p axis activating VEGFR2 signaling.** (A-B) Immunoblot of VEGFR2, p-VEGFR2, PLCγ and p-PLCγ of HUVECs incubated with conditioned medium from *DMDRMR* KD 786-O cells transfected with miR-378a-5p inhibitor (A) and *DMDRMR* OE 769-P cells transfected with miR-378a-5p mimic (B). (C-G) Cell proliferation assay assessing the IC_50_ in 786-O cells transfected with indicated vectors (C), and in 769-P cells transfected with indicated vectors (D) following treatment with sunitinib for 5 days. (E) Cell proliferation assay assessing the IC_50_ in *DMDRMR* OE 786-O cells following treatment with pazopanib. (F) The tumor weights of *DMDRMR* KD and control 786-O cells in nude mice with PBS or sunitinib treatment. The results are presented as mean ± SEM. * *p* < 0.05, ** *p* < 0.01, and ****p* < 0.001. ns, not significant.

**
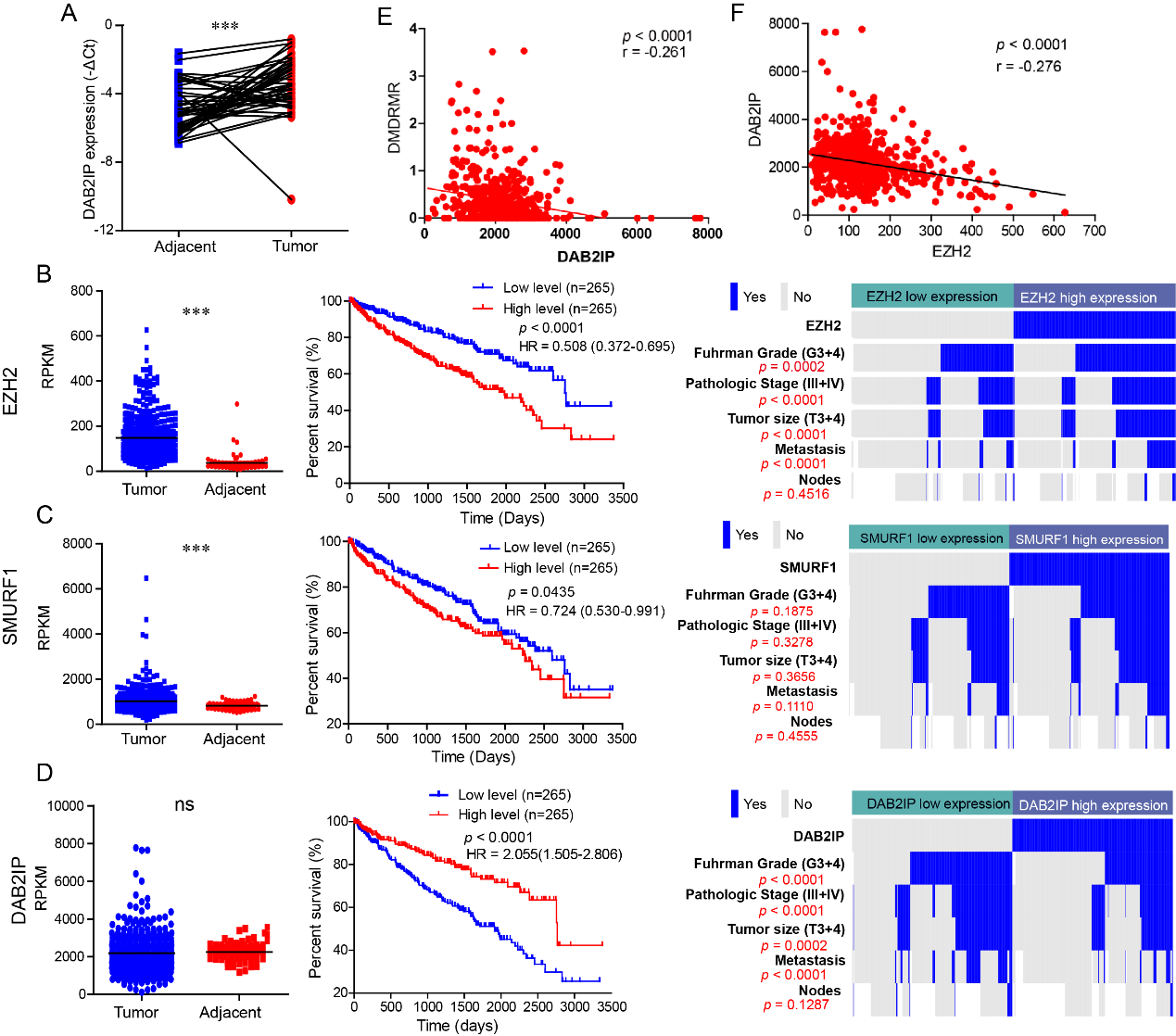
Figure S9. The clinical relevance of the *EZH2*, *SMURF1* and *DAB2IP* mRNA levels.** (A) The qRT-PCR analysis of *DAB2IP* express levels in 48 paired ccRCC and adjacent tissues. (B-D) Left: The mRNA levels of *EZH2* (B), *SMURF1* (C) and *DAB2IP* (D) in tumor and adjacent tissues across TCGA ccRCC cohort. Middle: Kaplan-Meier survival analysis of overall survival based on their mRNA levels in the TCGA ccRCC patients, respectively. Right: Heatmap plots illustrate the association of clinical characters with *EZH2* (B), *SMURF1* (C) and *DAB2IP* (D) high- and low-levels, respectively. The median expression levels were used as cut-off value. (E-F) Correlation analysis between *DAB2IP* and *DMDRMR* expression levels (E), *DAB2IP* and *EZH2* expression levels (F) in TCGA ccRCC and adjacent tissues. The results are presented as mean ± SD. *** *p* < 0.001. ns, not significant.

**
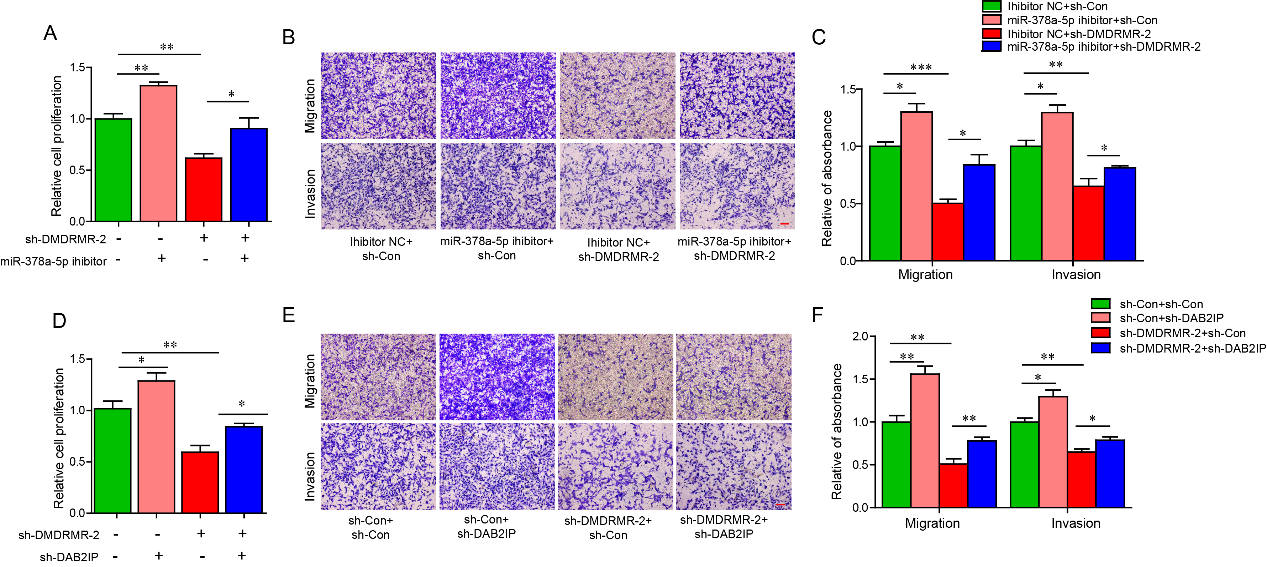
Figure S10. *DMDRMR* promotes cell proliferation, migration and invasion of ccRCC through its ceRNA.** (A) Cell proliferation assay assessing *DMDRMR* KD 786-O cells transfected with miR-378a-5p inhibitor on the fifth day. (B-C) Representative micrographs (B) and quantifications (C) of the abovementioned cells in the Matrigel-coated or noncoated Transwell assays (scale bars, 200 µm). (D) Cell proliferation assay assessing *DMDRMR* KD 786-O cells with DAB2IP KD on the fifth day. (E-F) Representative micrographs (E) and quantifications (F) of the abovementioned cells in the Matrigel-coated or noncoated Transwell assays (scale bars, 200 µm). Results are presented as the mean ± SEM. **p* < 0.05, ***p* <0.01, and ****p* < 0.001.


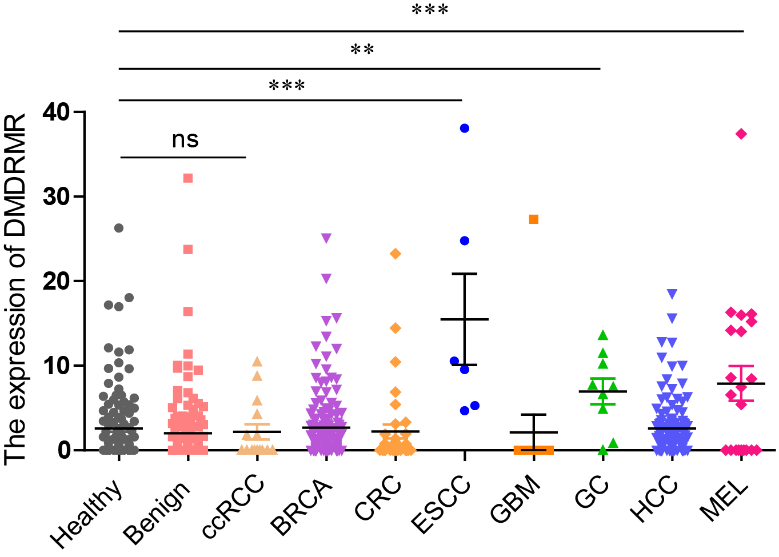


**Figure S11. The expression of extracellular vesicles *DMDRMR* derived from blood of human healthy and cancer cohorts.** BRCA, breast cancer; CRC, colorectal cancer; ESCC, esophageal squamous cell carcinoma; GBM, glioblastoma multiforme; GC, gastric cancer; HCC, hepatocellular carcinoma; MEL, melanoma. Results are presented as the mean ± SEM. ***p* <0.01, ****p* < 0.001. ns, no significant.

**Supplementary Tables**

**Table S1 Sequences of shRNA for KD.**

| **Targets** | **shRNA-Forward (5'-3')** | **shRNA-Reverse (5'-3')** |
| --- | --- | --- |
| sh-*DMDRMR*-1 | GATCCGGTATCGCCCAGATCTGAATCTTCAAGAGAGATTCAGATCTGGGCGATACCTTTTTTG | AATTCAAAAAAGGTATCGCCCAGATCTGAATCTCTCTTGAAGATTCAGATCTGGGCGATACCG |
| sh-*DMDRMR*-2 | GATCCTCGCCCAGATCTGAATCCACACTTCAAGAGAGTGTGGATTCAGATCTGGGCGATTTTTTG | AATTCAAAAAATCGCCCAGATCTGAATCCACACTCTCTTGAAGTGTGGATTCAGATCTGGGCGAG |
| sh-DAB2IP | GATCCGTAATGTAACTATCTCACCTATTCAAGAGATAGGTGAGATAGTTACATTACTTTTTTG | AATTCAAAAAAGTAATGTAACTATCTCACCTATCTCTTGAATAGGTGAGATAGTTACATTACG |
| sh-EZH2 | GATCCGTGCTGCTGCTCTCACCGCTGAGCGGATAATTCAAGAGATTATCCGCTCAGCGGTGAGAGCAGCAGCACTTTTTTG | AATTCAAAAAAGTGCTGCTGCTCTCACCGCTGAGCGGATAATCTCTTGAATTATCCGCTCAGCGGTGAGAGCAGCAGCAC  G |
| sh-SMURF1 | GATCCGCCCAGAGATACGAAAGAGATTTCAAGAGAATCTCTTTCGTATCTCTGGGCTTTTTTG | AATTCAAAAAAGCCCAGAGATACGAAAGAGATTCTCTTGAAATCTCTTTCGTATCTCTGGGCG |

**Table S2 List of qRT-PCR primers.**

| **Genes** | **Forwards (5'-3')** | **Reverses (5'-3')** |
| --- | --- | --- |
| *DMDRMR* | ATGCTGGAAGAACGAGCCTT | GGCCCTGGTCCTAGGTCATA |
| GAPDH | GGAGCGAGATCCCTCCAAAAT | GGCTGTTGTCATACTTCTCATGG |
| VEGFA | CTTGCCTTGCTGCTCTACCT | GCAGTAGCTGCGCTGATAGA |
| EZH2 | AAGGAGTTTGCTGCTGCTCT | ATTAATGGTGGGGGTGCTGG |
| SMURF1 | GTCCCGCTCCAAGGCTTC | GATGCTTTTGGTCTGGTGGC |
| DAB2IP | CTCCACCAAGAAGCTGGAGG | CTGCGTGGTCCTTCTTCAGT |
| H19 | CAGGAATCGGCTCTGGAAGG | TGGCCATGAAGATGGAGTCG |
| EMS | TCCACCTAGGACTACAGGCT | GTGGCTTCTCGGCTGAATCC |

**Table S3 List of ChIP qRT-PCR primers.**

| **Regions** | **Forwards (5'-3')** | **Reverses (5'-3')** |
| --- | --- | --- |
| The first region of DAB2IP promoter | CCTGCTTTCTGTTTCCTTCTCCTG | TTGAACCACCTCCTCCTCCCTCTC |
| The second region of DAB2IP promoter | TCACCTCTCCACTCTCAGGG | CAGGTTGAACCACCTCCTCC |

**Table S4 List of primary antibodies.**

| **Antibodies** | **Manufacturer** | **Application** |
| --- | --- | --- |
| CD31 | Abcam, #ab28364 | 1:400 for IHC |
| GAPDH | Proteintech, #60004-1-Ig | 1:5000 for WB |
| ɑTubulin | Proteintech, #66031-1-Ig | 1:5000 for WB |
| VEGFA | Abcam, #ab46154 | 1:1000 for WB,1:400 for IHC |
| EZH2 | Abcam, #ab191080 | 1:2000 for WB |
| SMURF1 | Abcam, #ab57573 | 1:2000 for WB |
| EZH2 | Merckmillipore, #17-662 | 2ug for ChIP |
| H3K27me3 | Merckmillipore, #17-622 | 2ug for ChIP |
| FLAG | CST, #14793 | 1:2000 for WB |
| IGG | Beyotime, #A7016 | 5ug for RIP |
| GFP | Abcam, #ab290 | 5ug for RIP |
| Ago2 | Merckmillipore, #03-110 | 5ug for RIP |
| p-VEGFR2 | CST, #3817 | 1:1000 for WB |
| VEGFR2 | Abcam, # ab2349 | 1:2000 for WB |
| p-PLCγ | CST, #14008s | 1:2000 for WB |
| PLCγ | CST, #2822s | 1:2000 for WB |
| HA | CST, #3724 | 1:2000 for WB |
| DAB2IP | Abcam, #ab87811 | 1:2000 for WB, 3ug for IP |

**References**

1. Livak KJ, Schmittgen TDJm. Analysis of relative gene expression data using real-time quantitative PCR and the 2− ΔΔCT method. 2001;**25**(4):402-8.

2. Patrone G, Puppo F, Cusano R, Scaranari M, Ceccherini I, Puliti A*, et al.* Nuclear run-on assay using biotin labeling, magnetic bead capture and analysis by fluorescence-based RT-PCR. 2000;**29**(5):1012-7.
