# Supplementary material for: DMDRMR promotes angiogenesis via antagonizing DAB2IP in clear cell renal cell carcinoma
Source: Cell Death Dis. 2022 May 13;13(5):456. doi: 10.1038/s41419-022-04898-3 (PMC9106801; doi:10.1038/s41419-022-04898-3)

Figure 1B

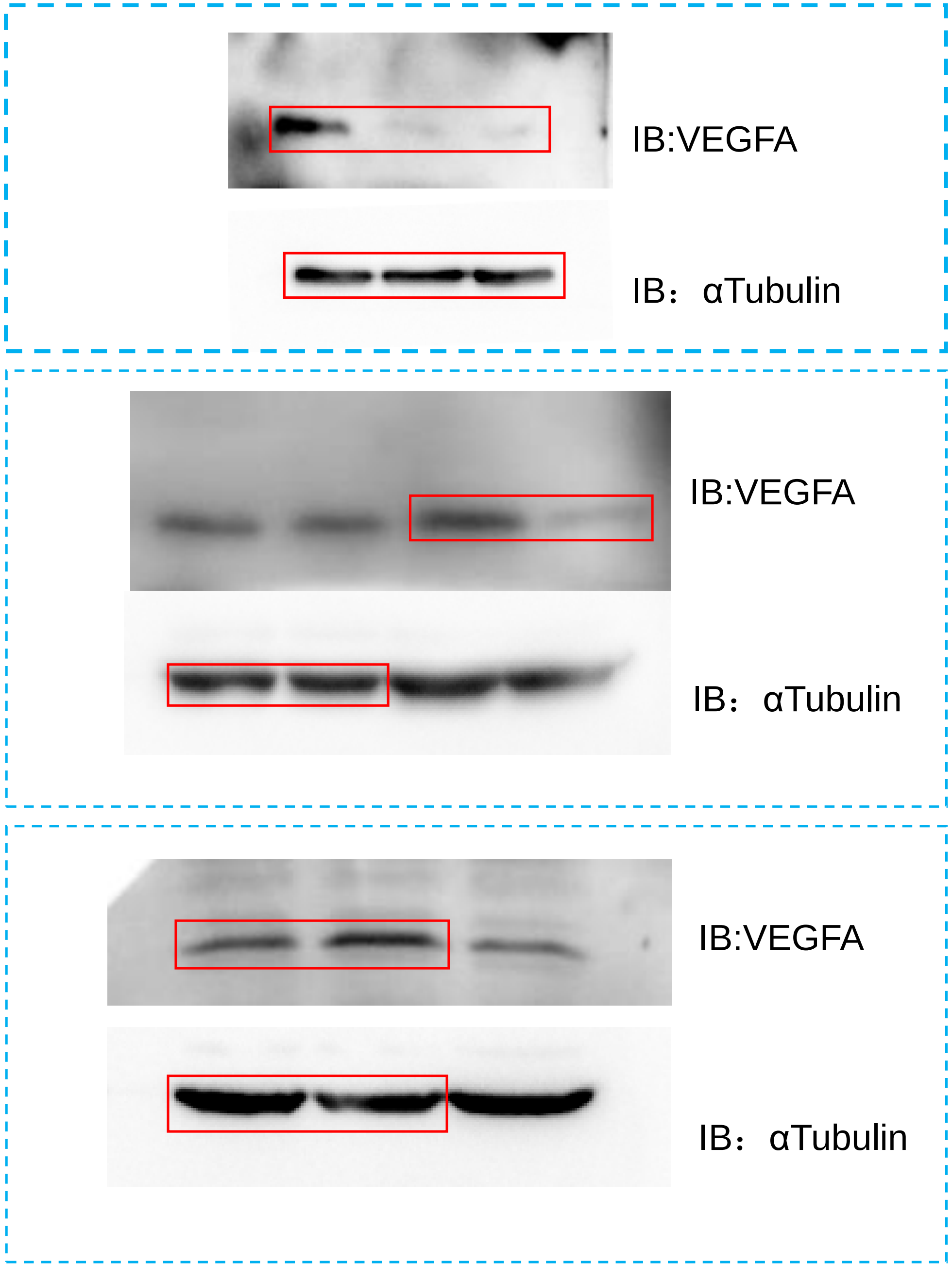

IB:VEGFA
IB： αTubulin
IB:VEGFA
IB： αTubulin
IB:VEGFA
IB： αTubulin

Figure 4E

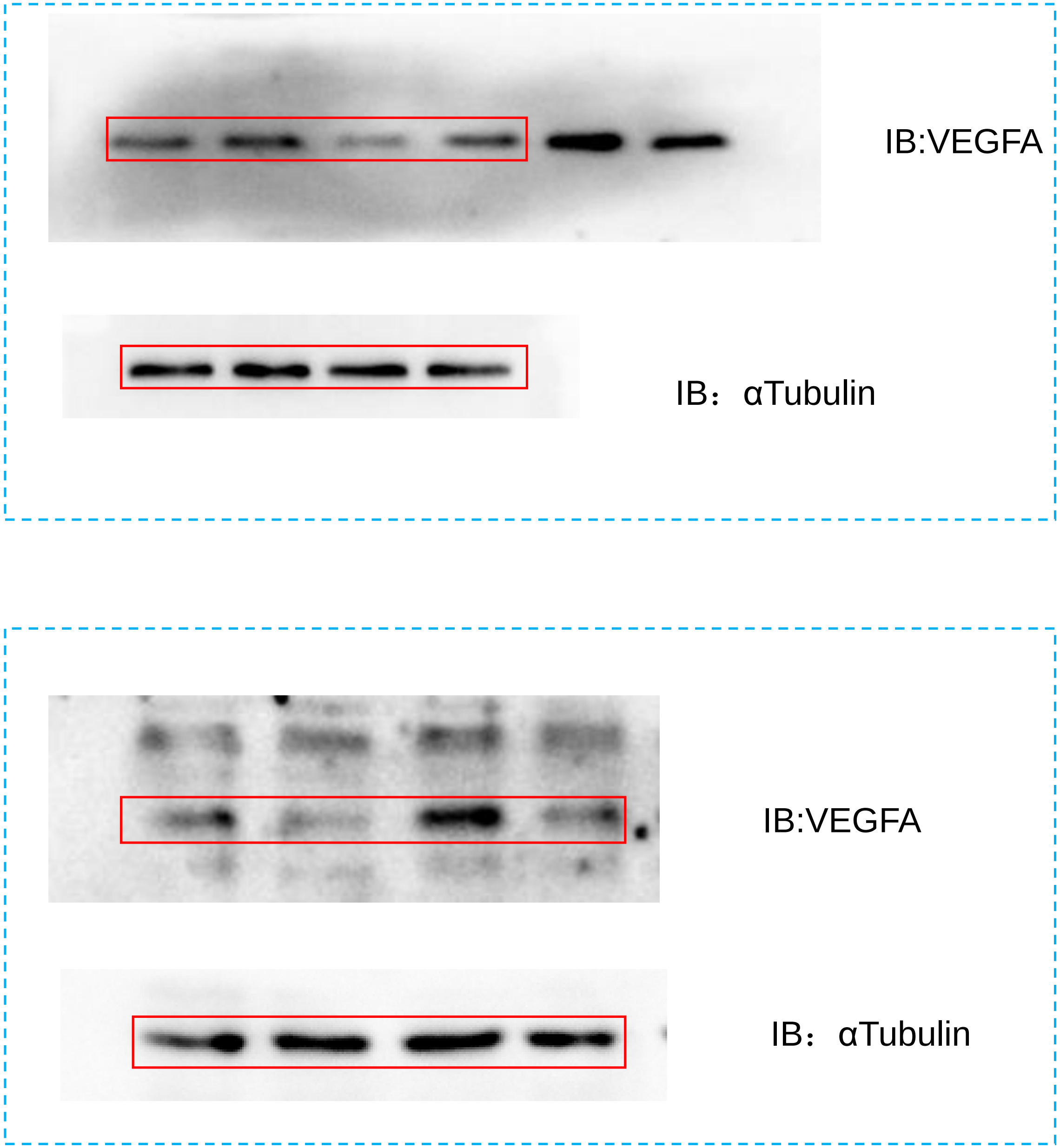

Figure 5G

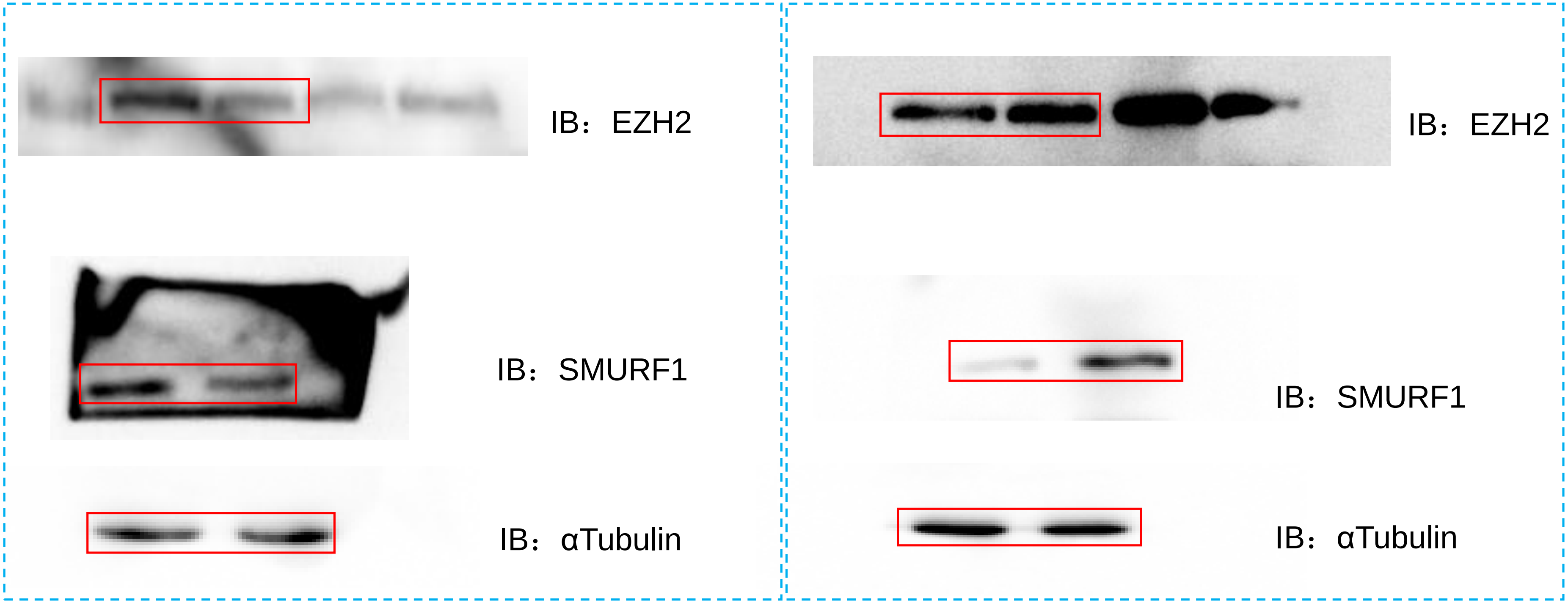

Figure 5H

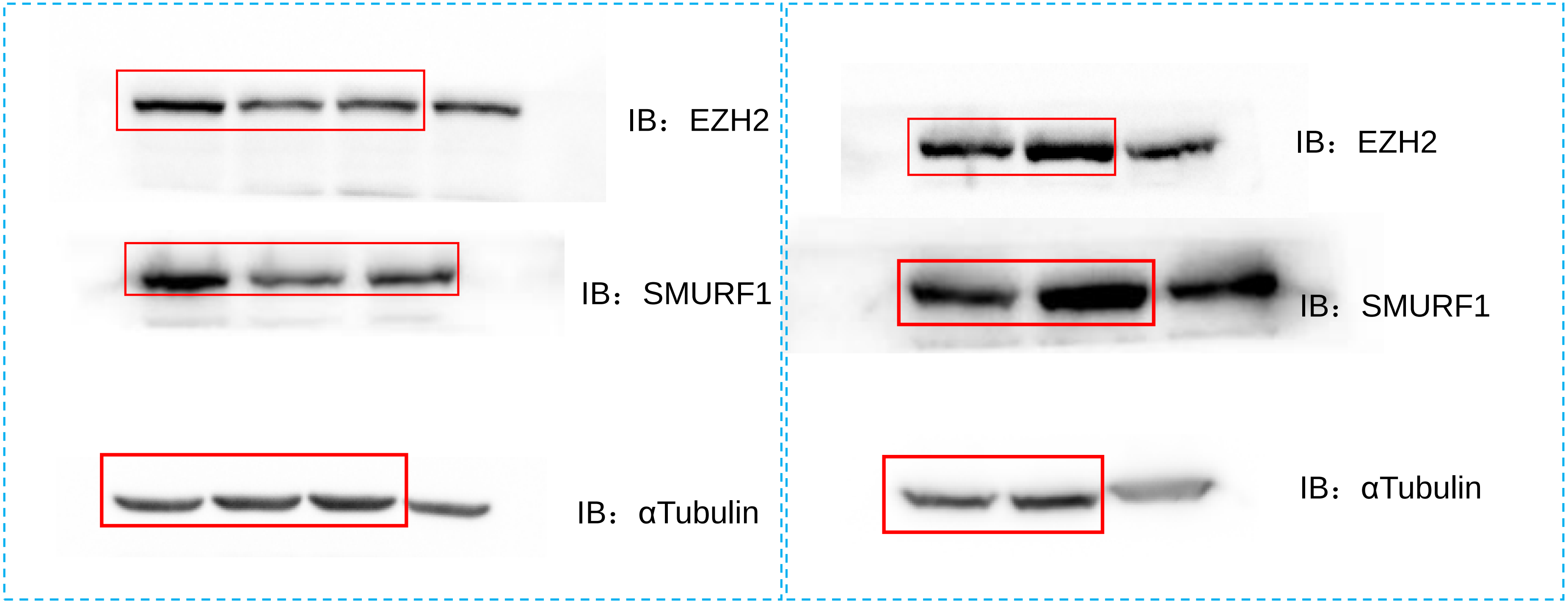

Figure 6A

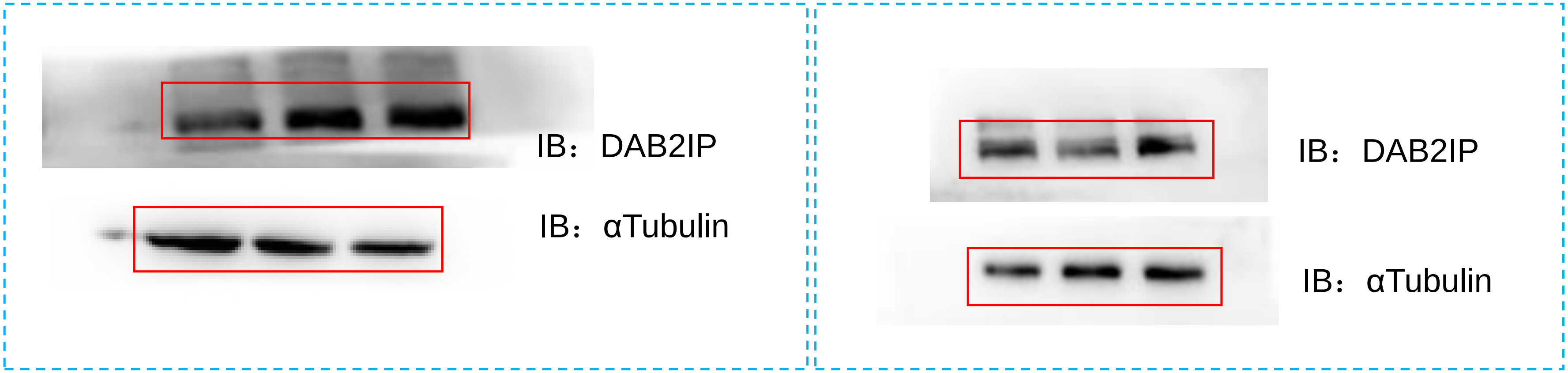

Figure 6C

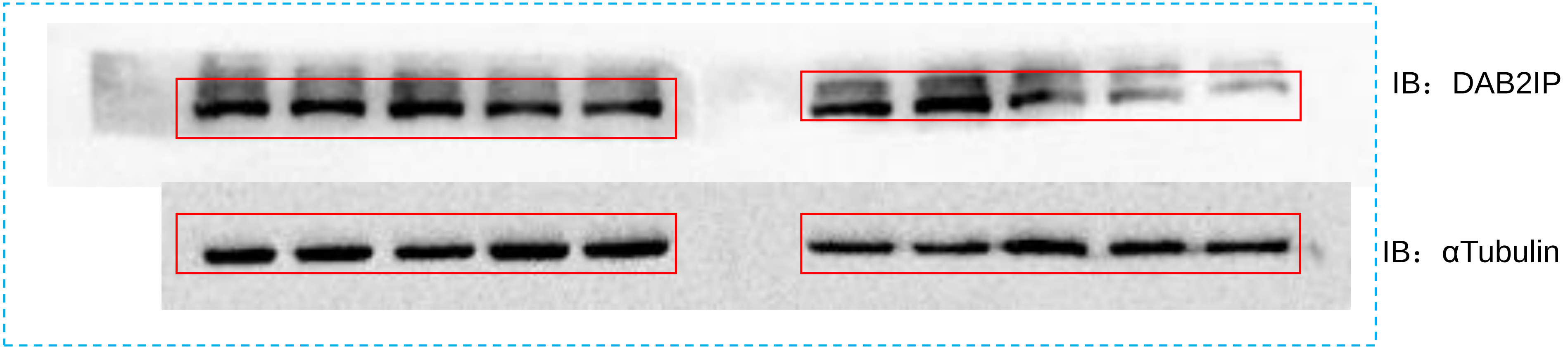

Figure 6D

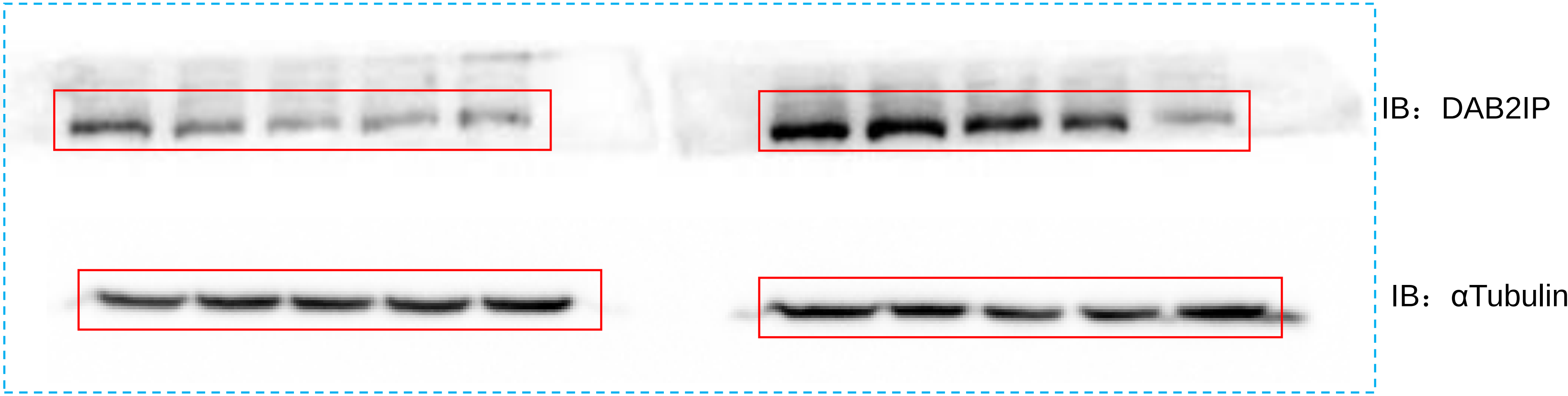

Figure 6E

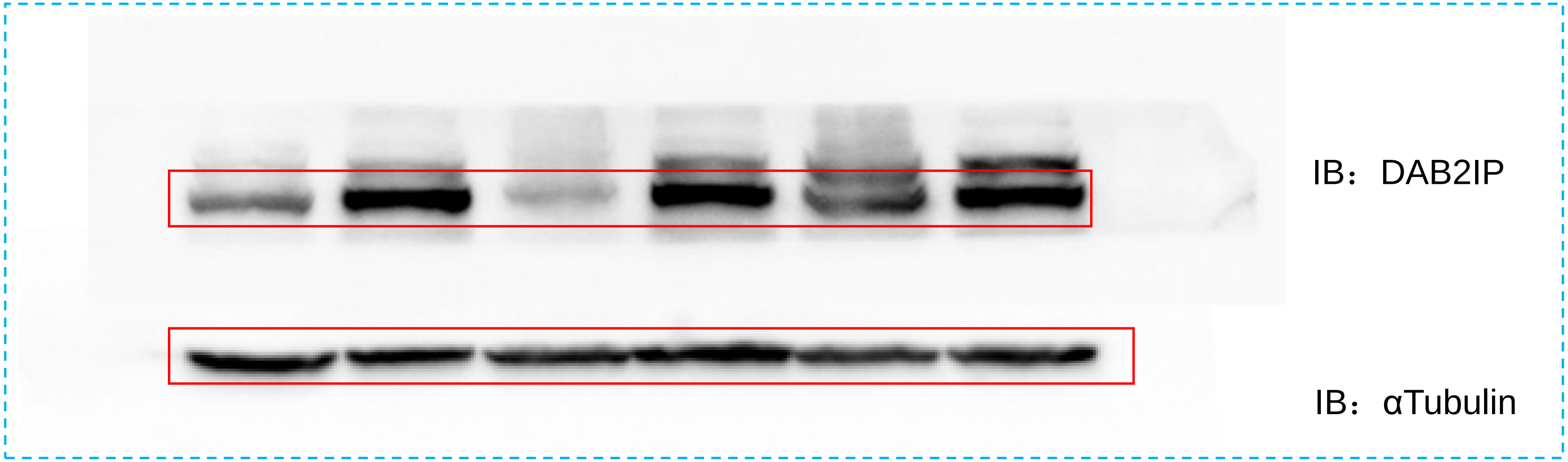

Figure 6F

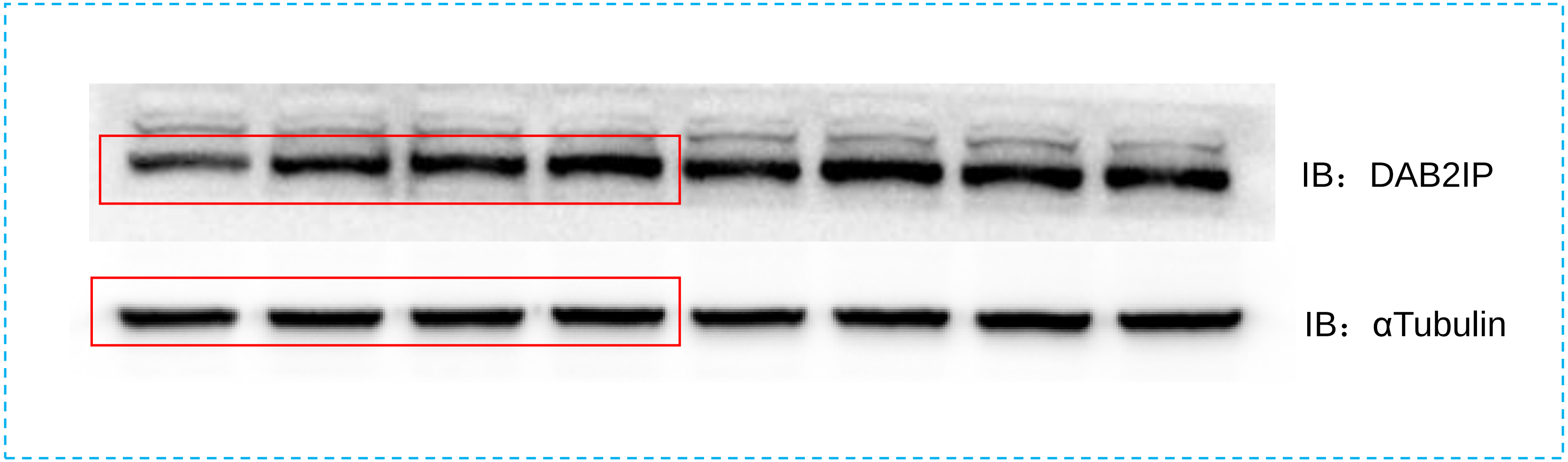

Figure 6G

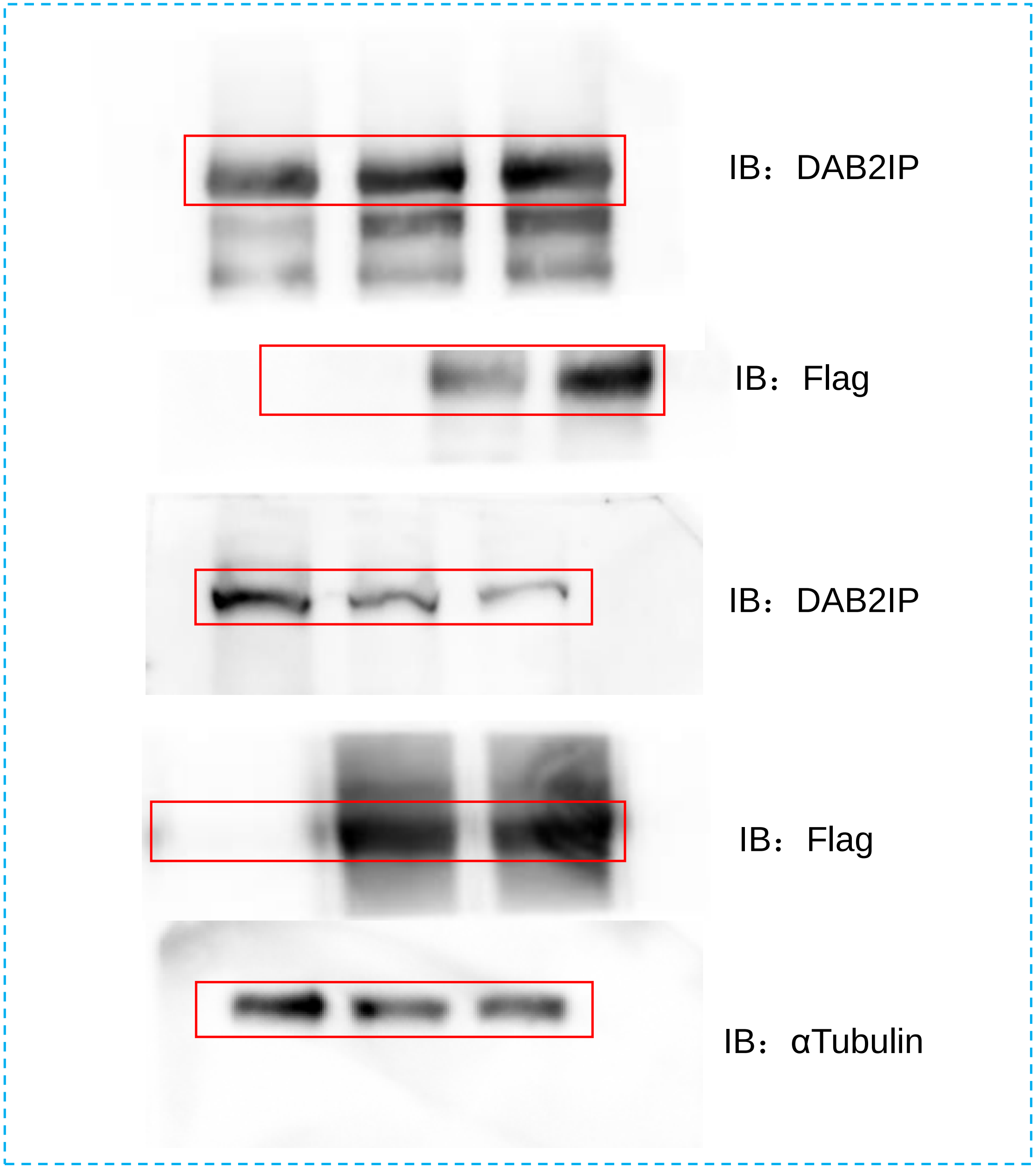

Figure 6H

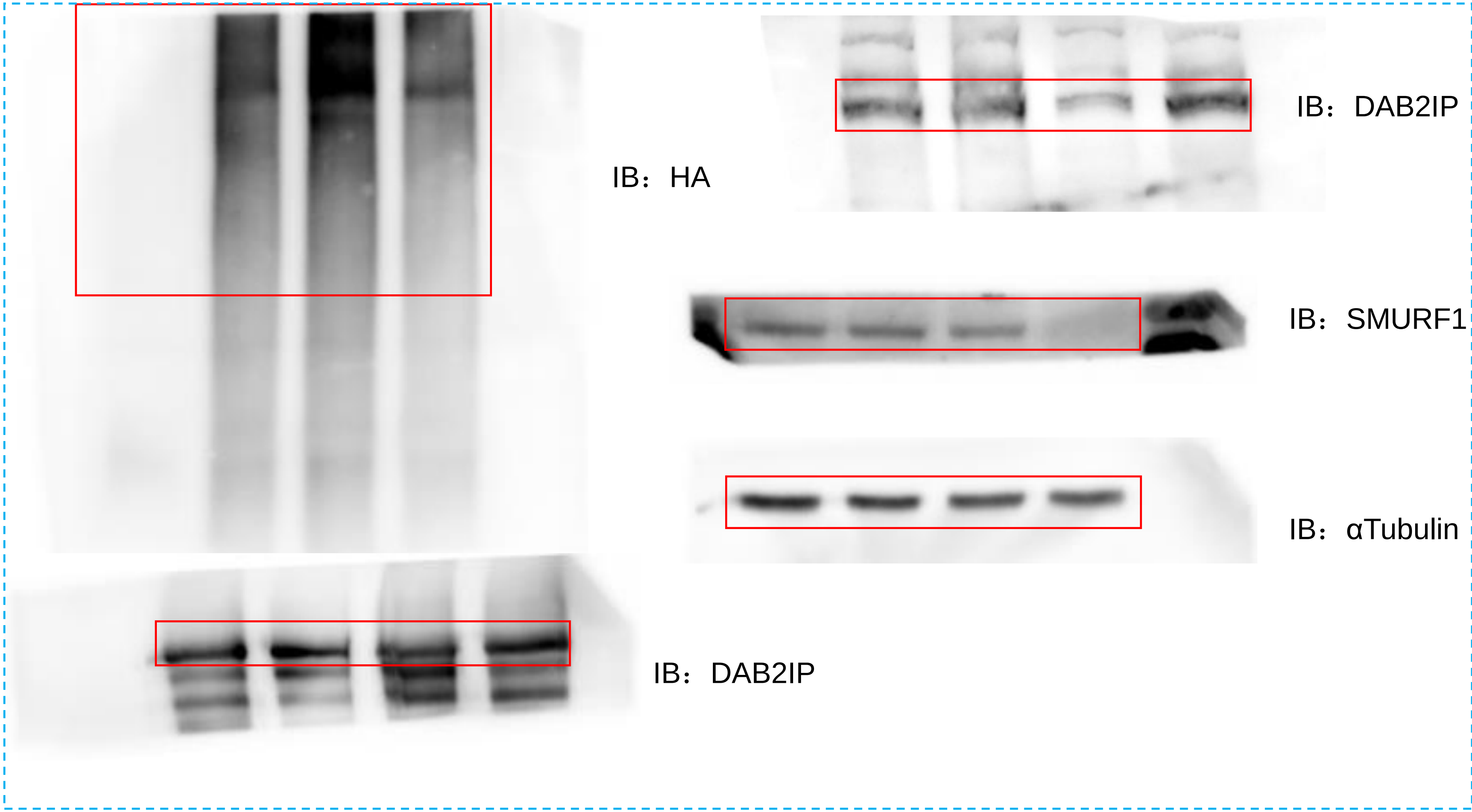

Figure 6I

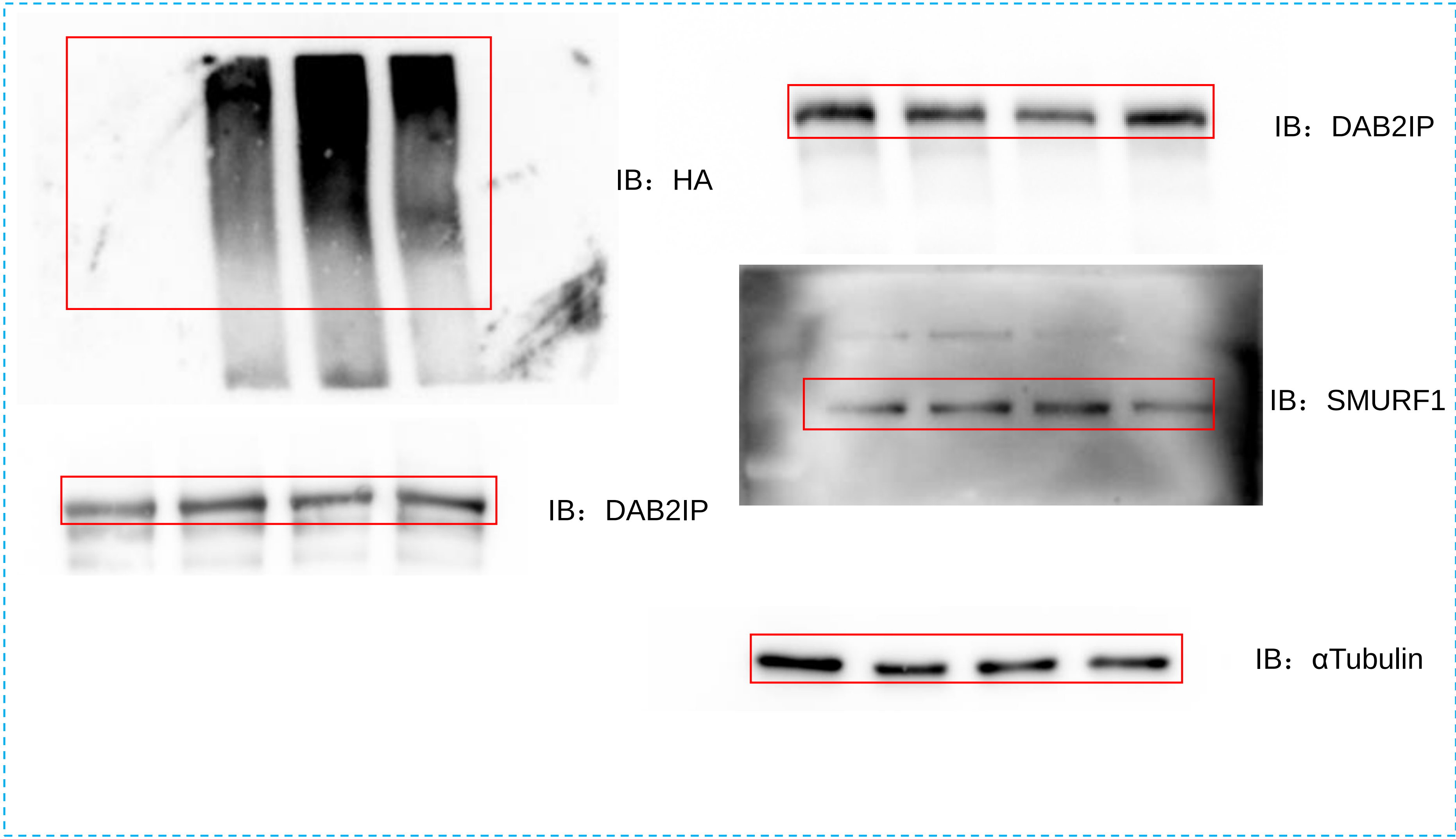

Figure 6J

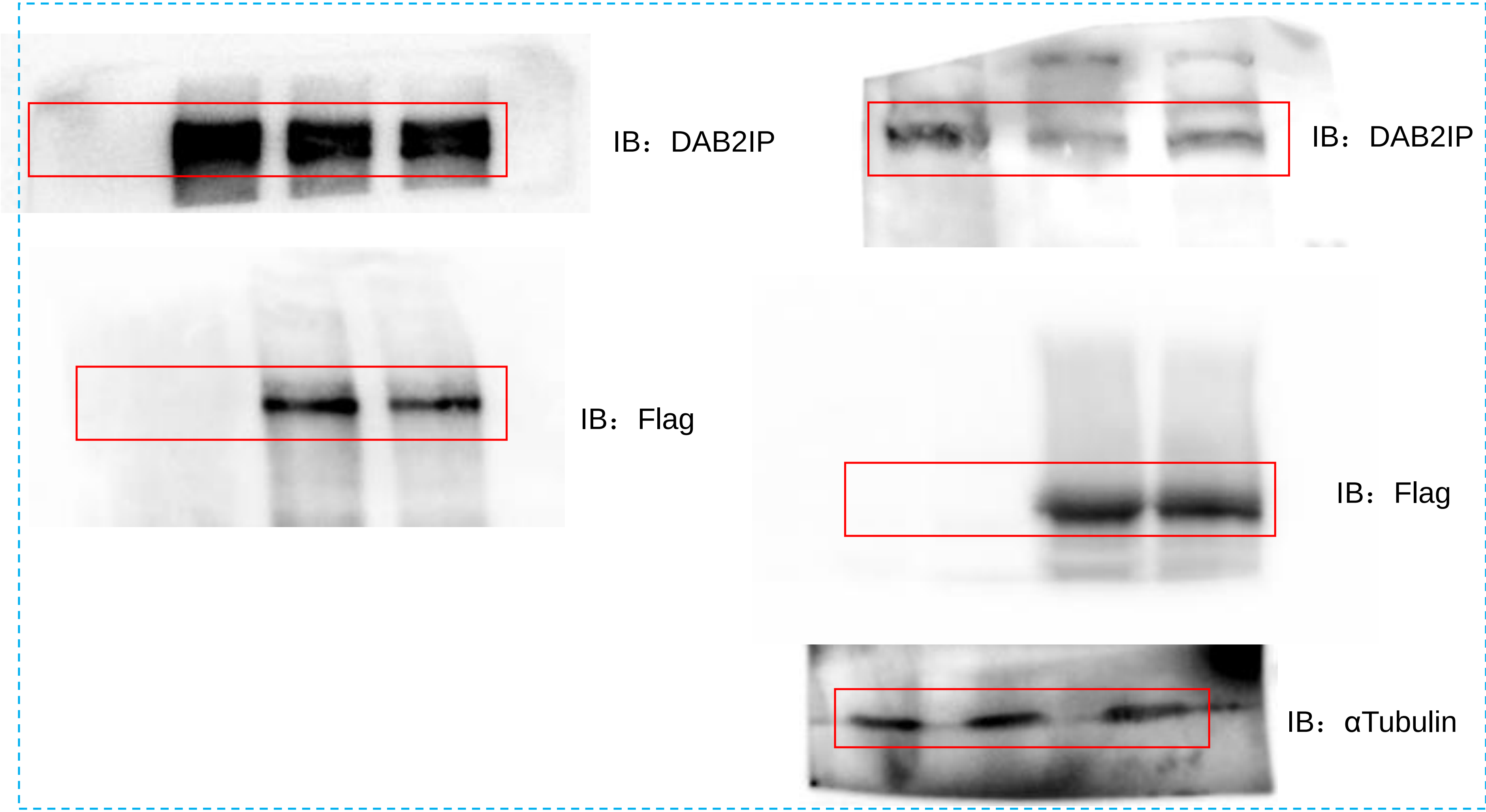

Figure 6K

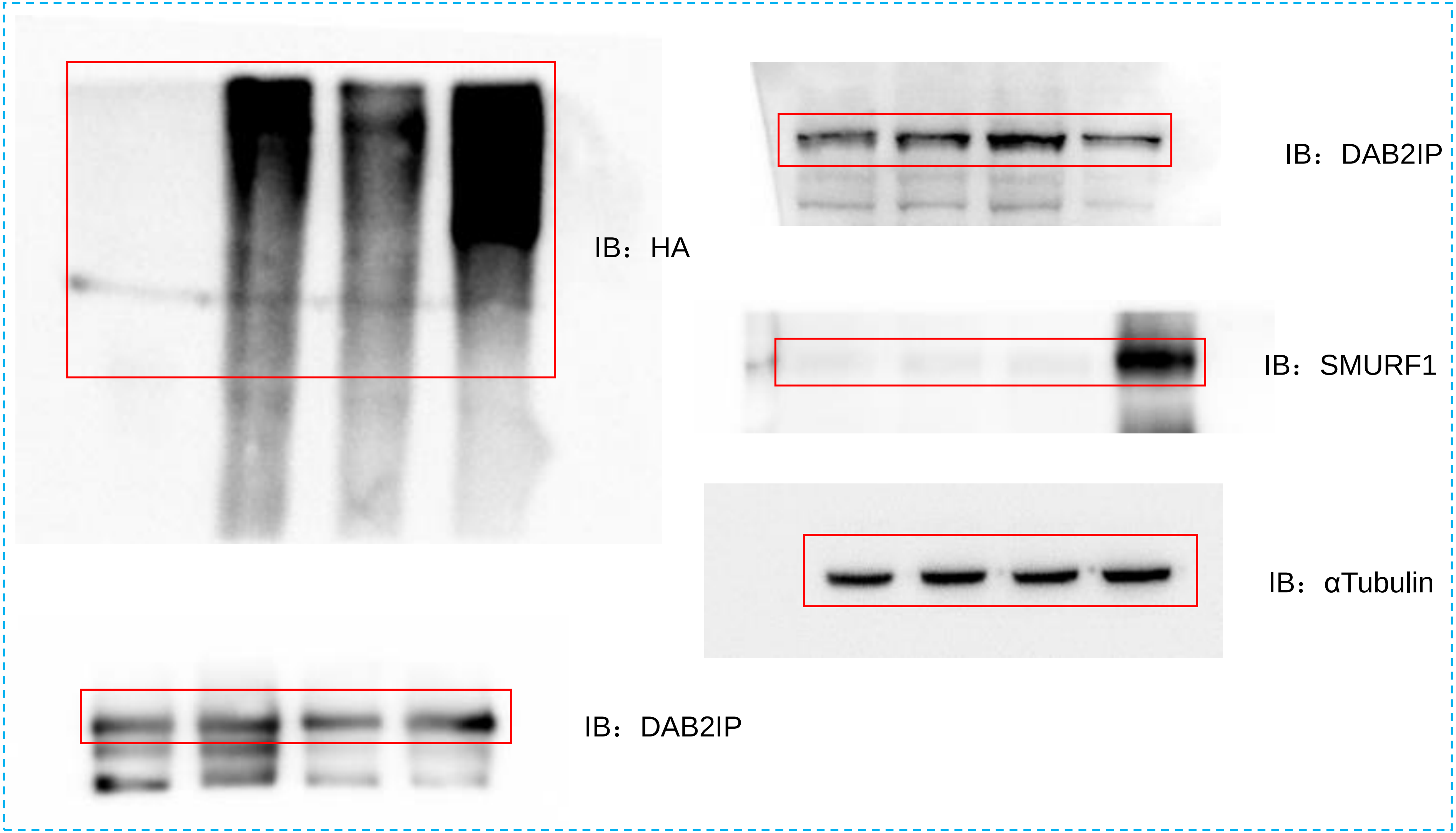

Figure 6L

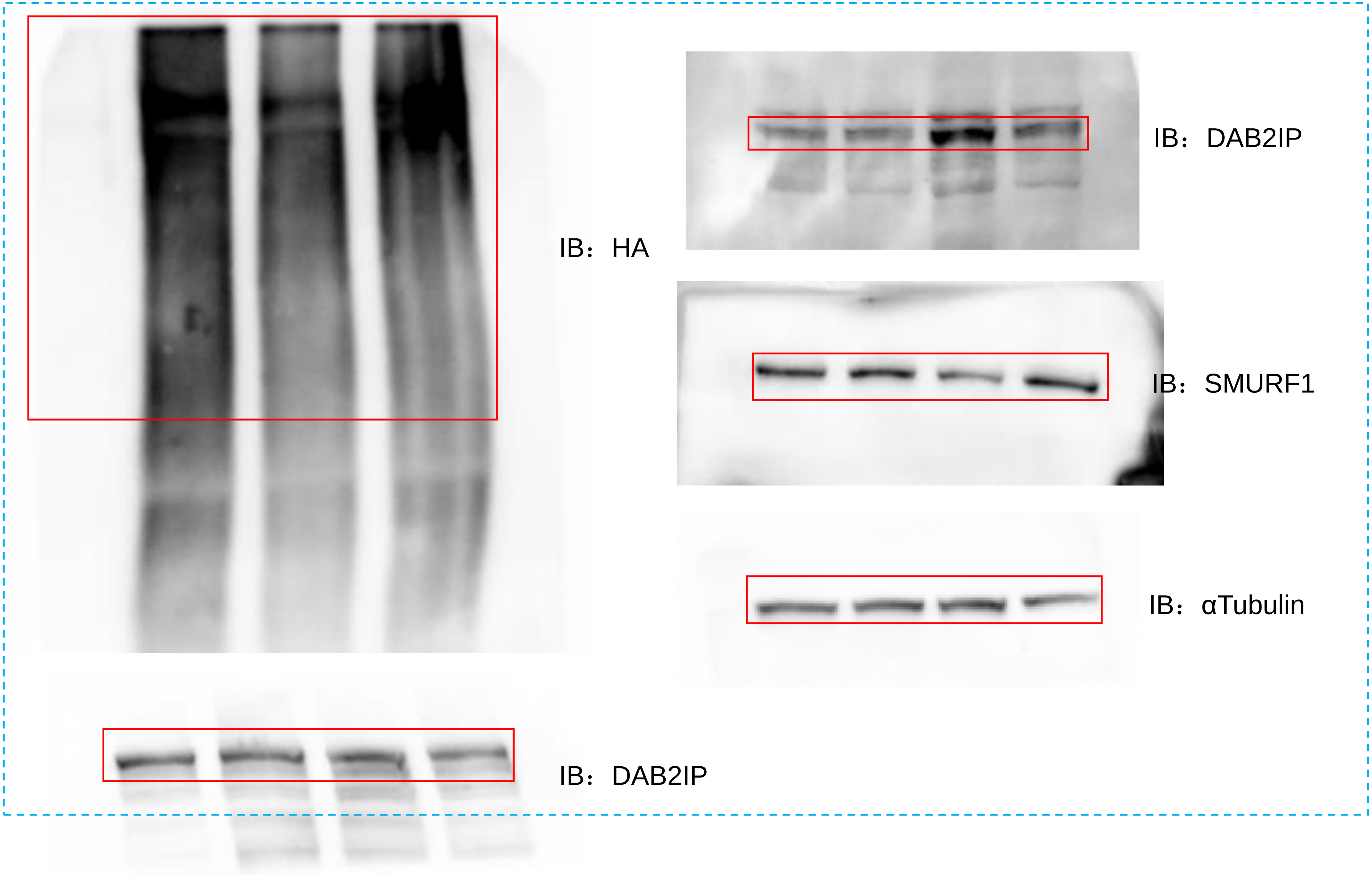

Figure 7C

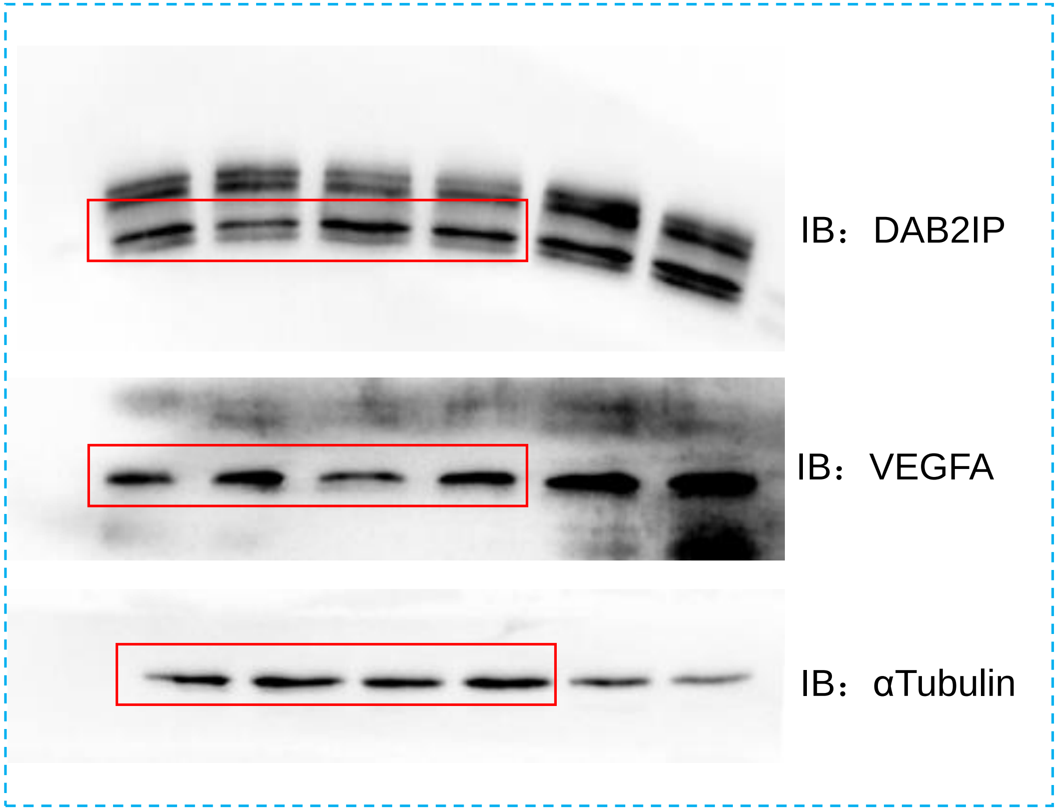

Figure 7D

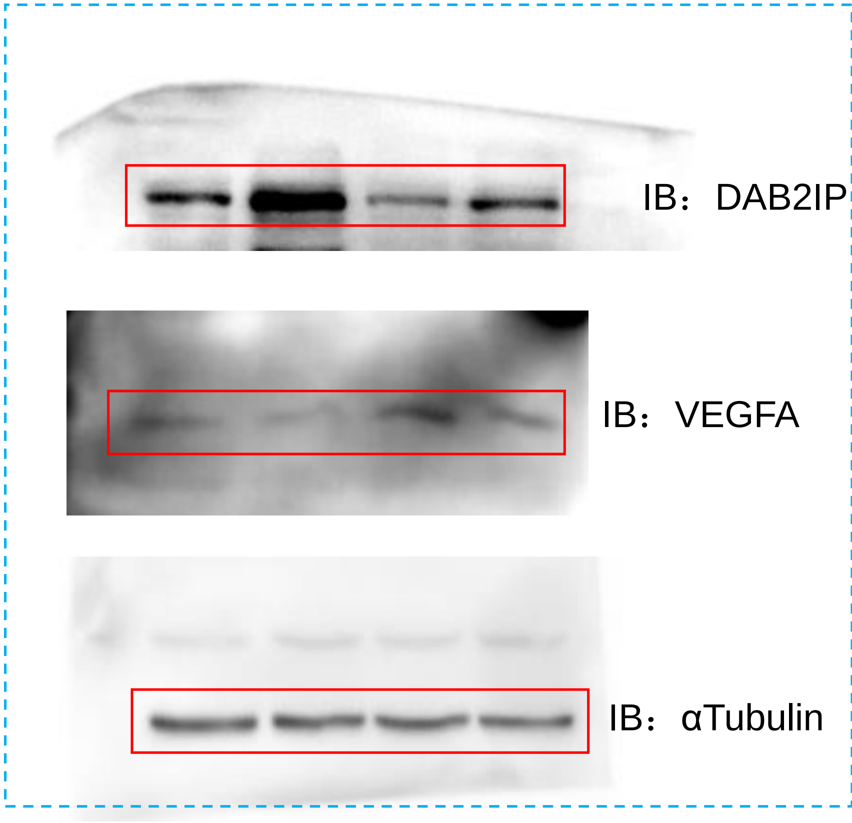

Figure 7E

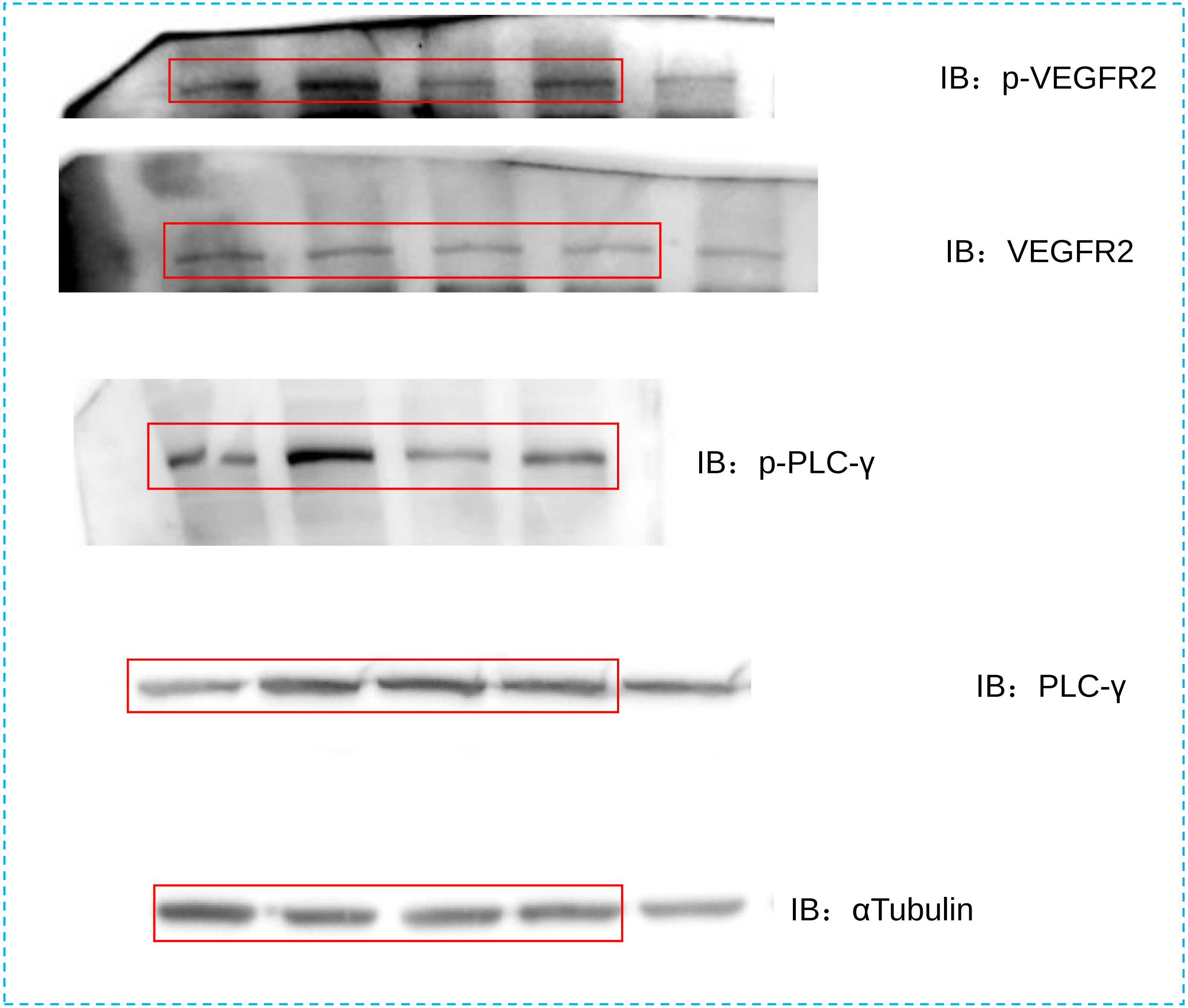

Figure 7F

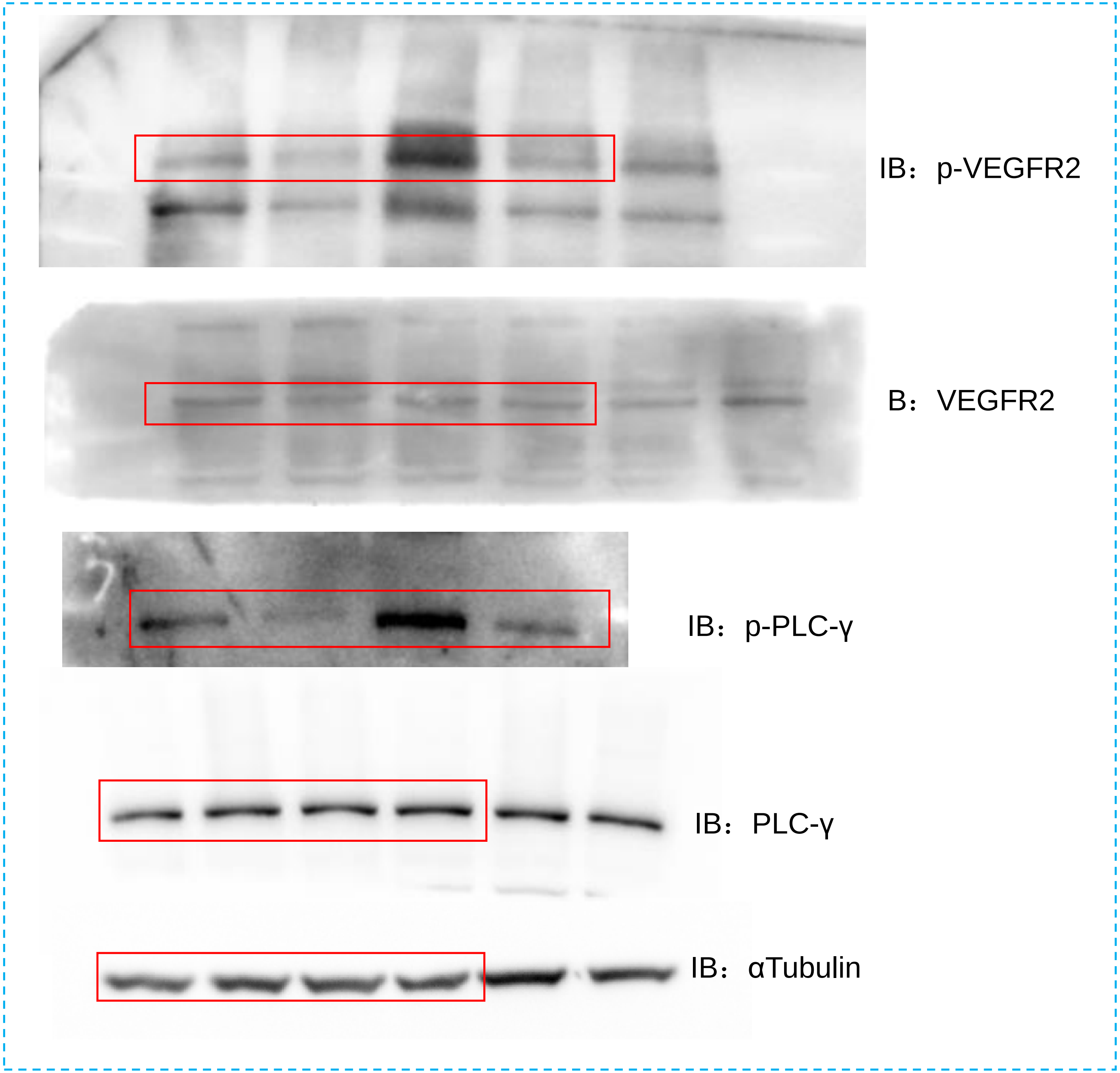

Figure S5C

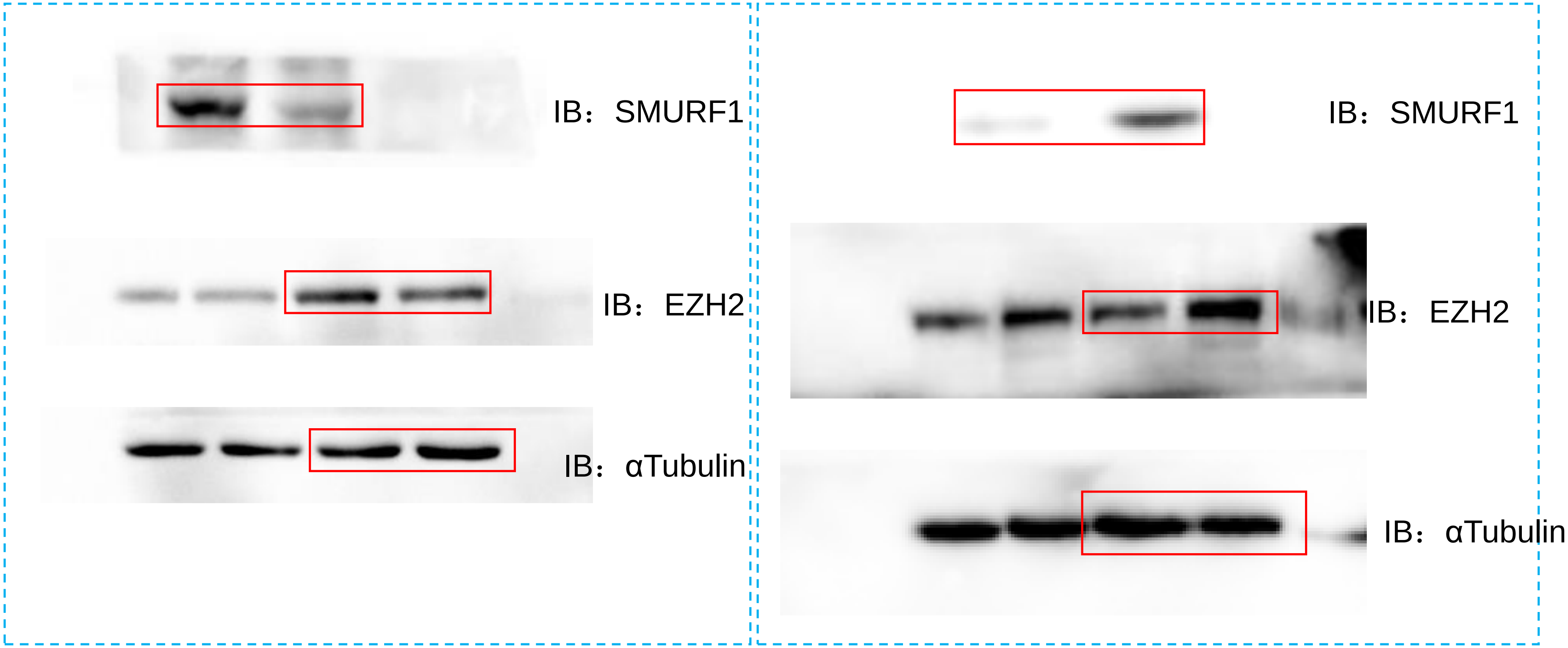

Figure S6B

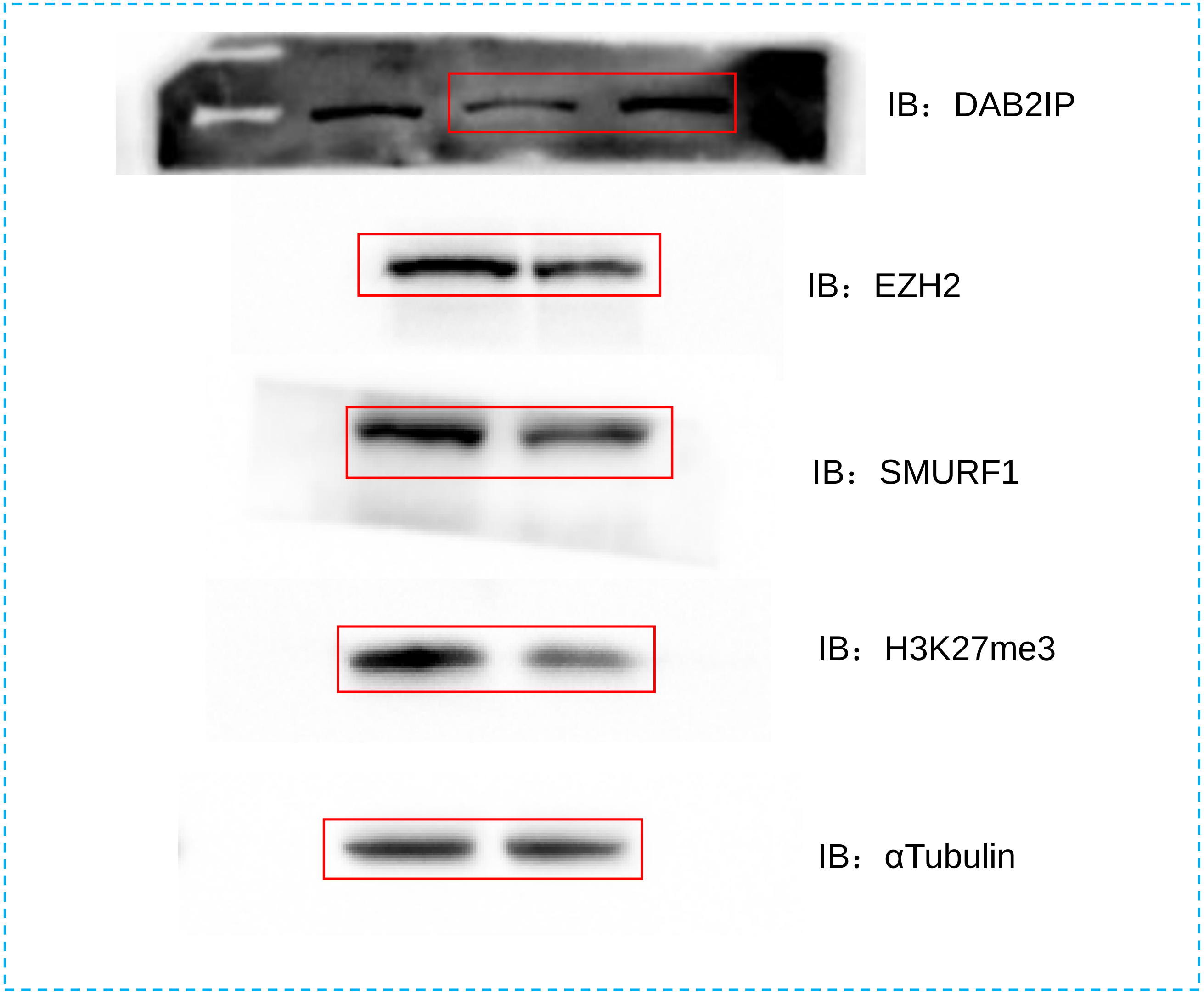

Figure S6D

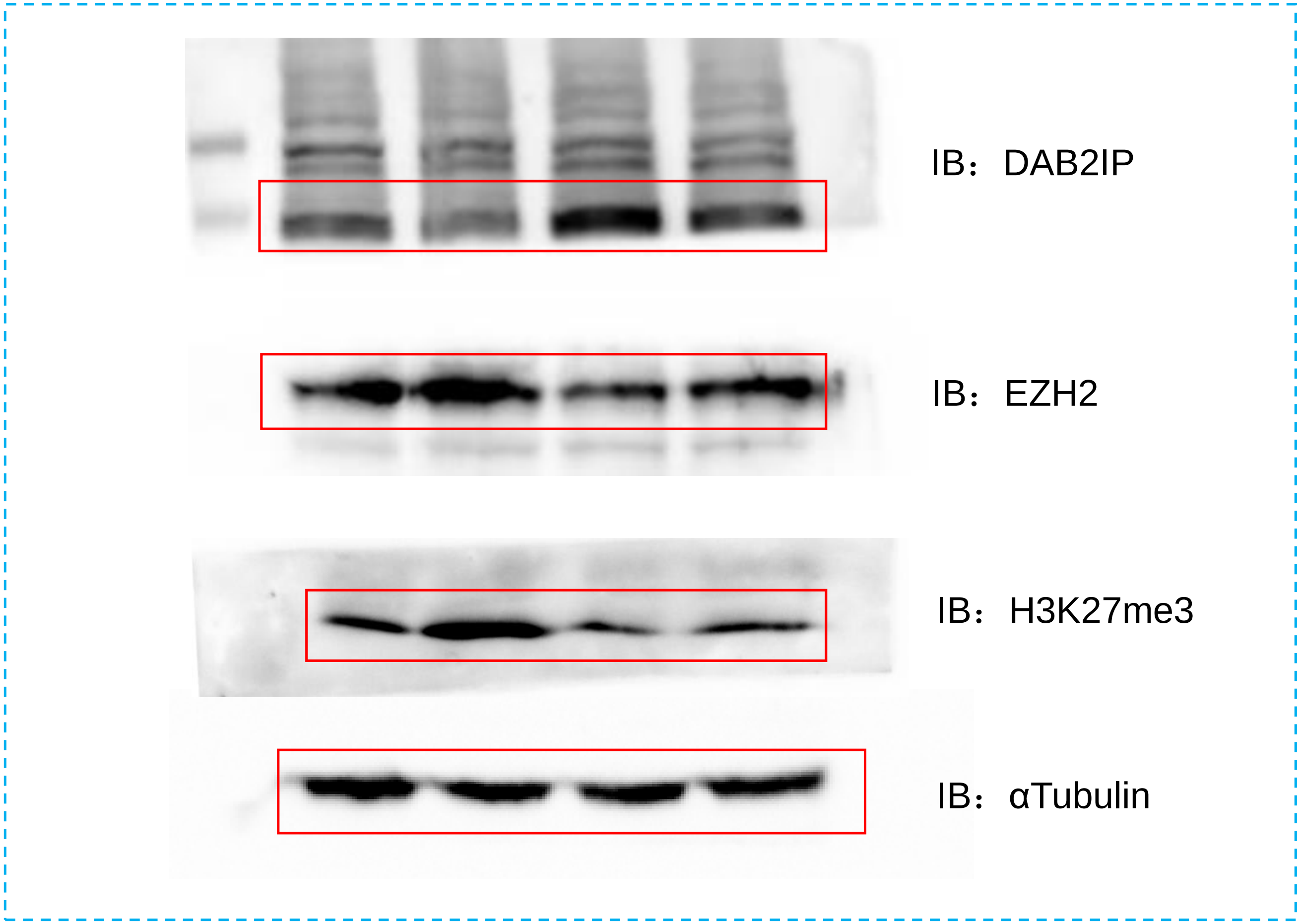

Figure S6E

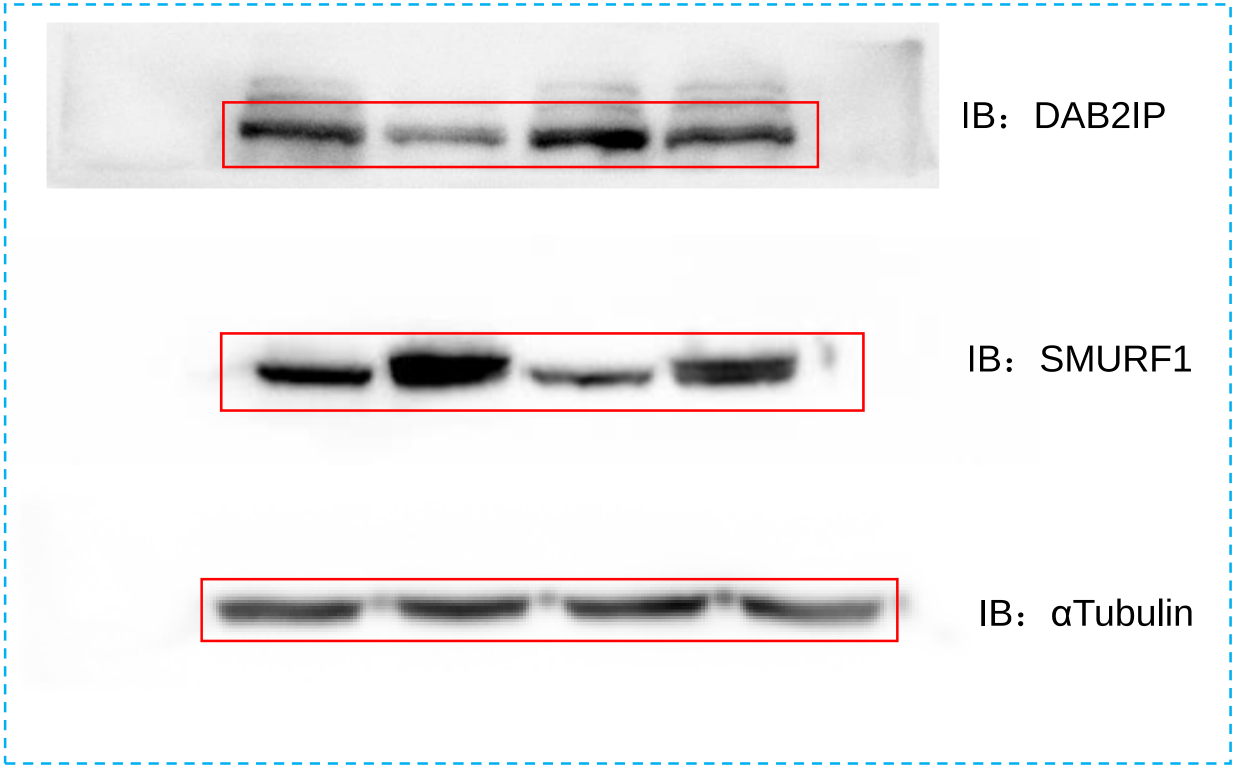

Figure S6F

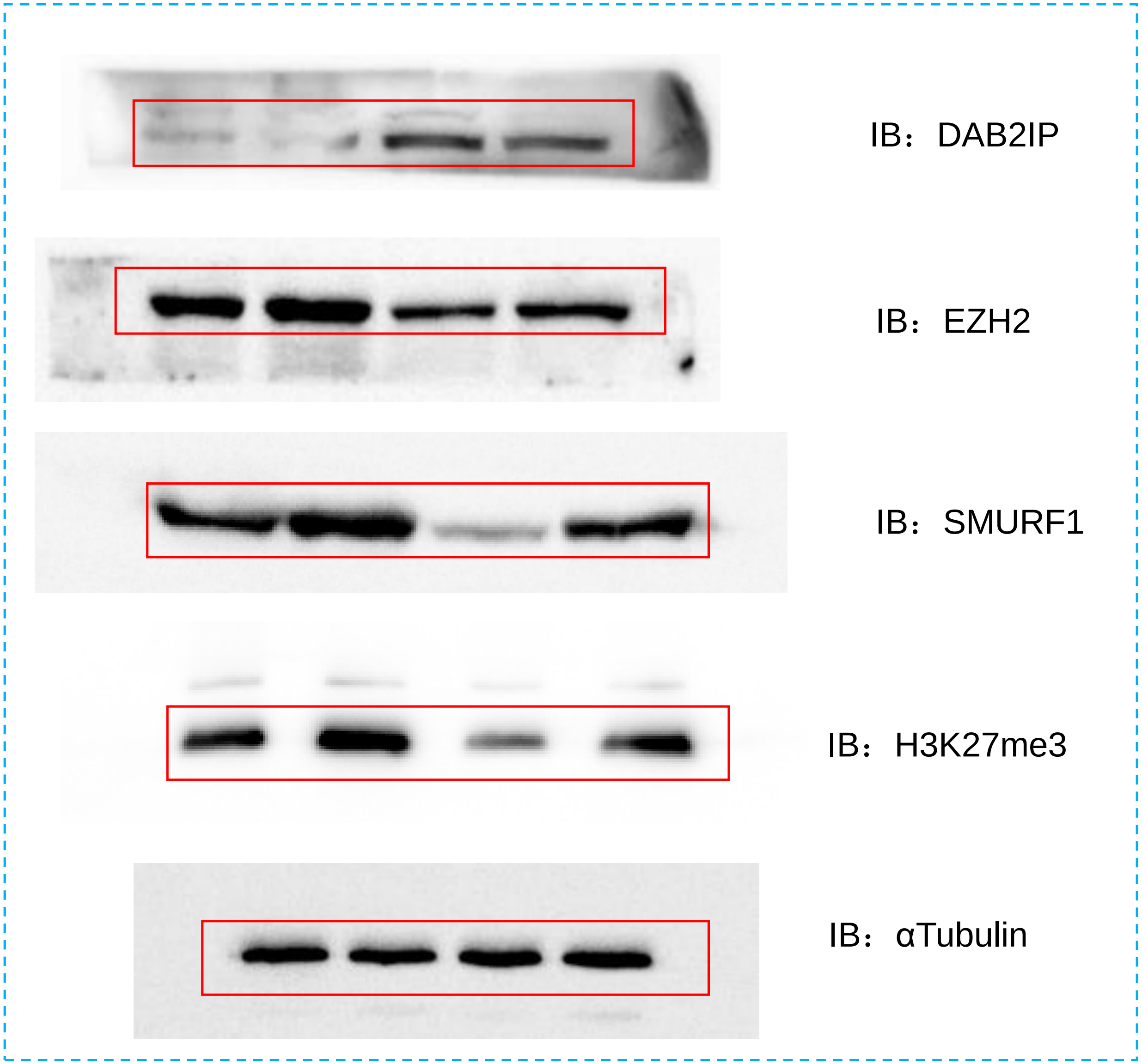

Figure S7A

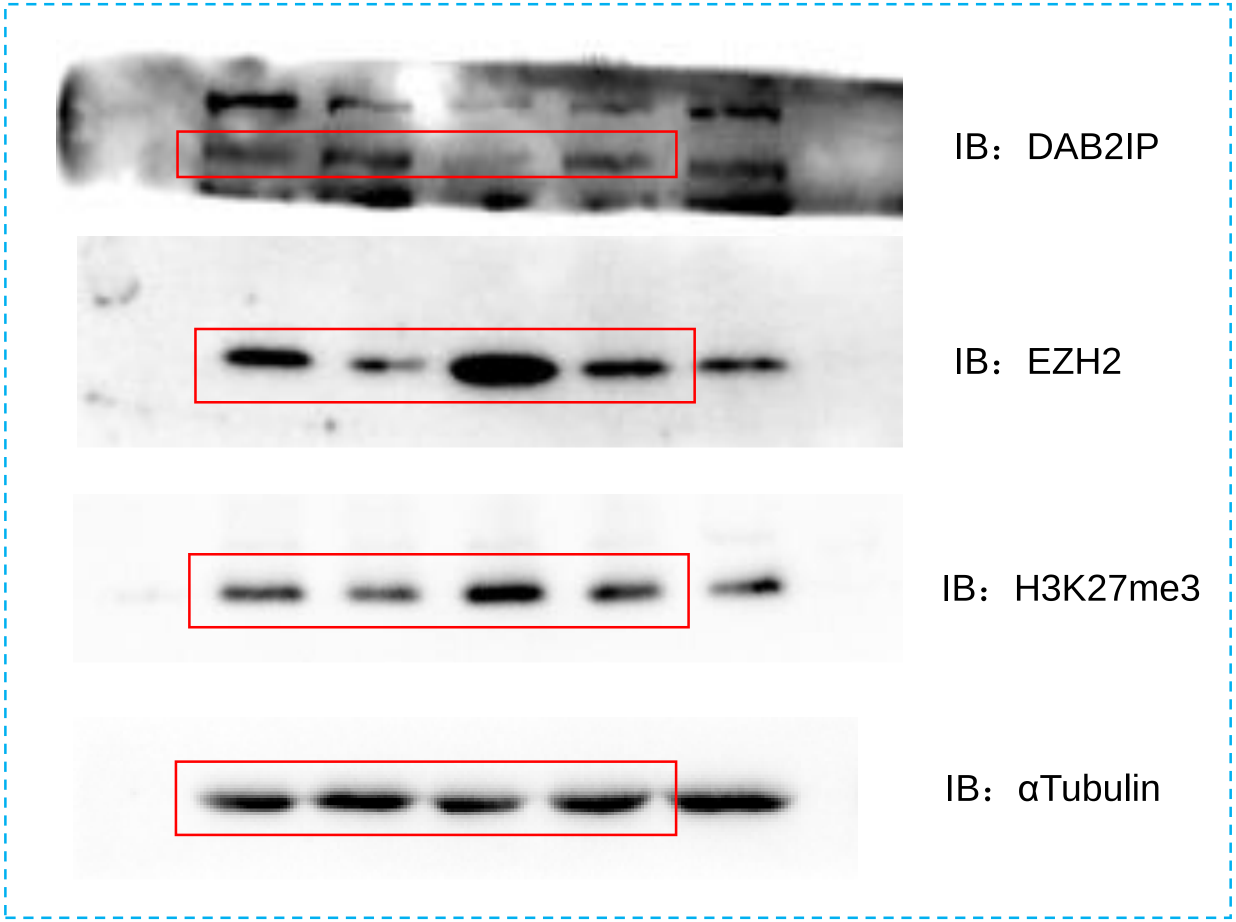

Figure S7B

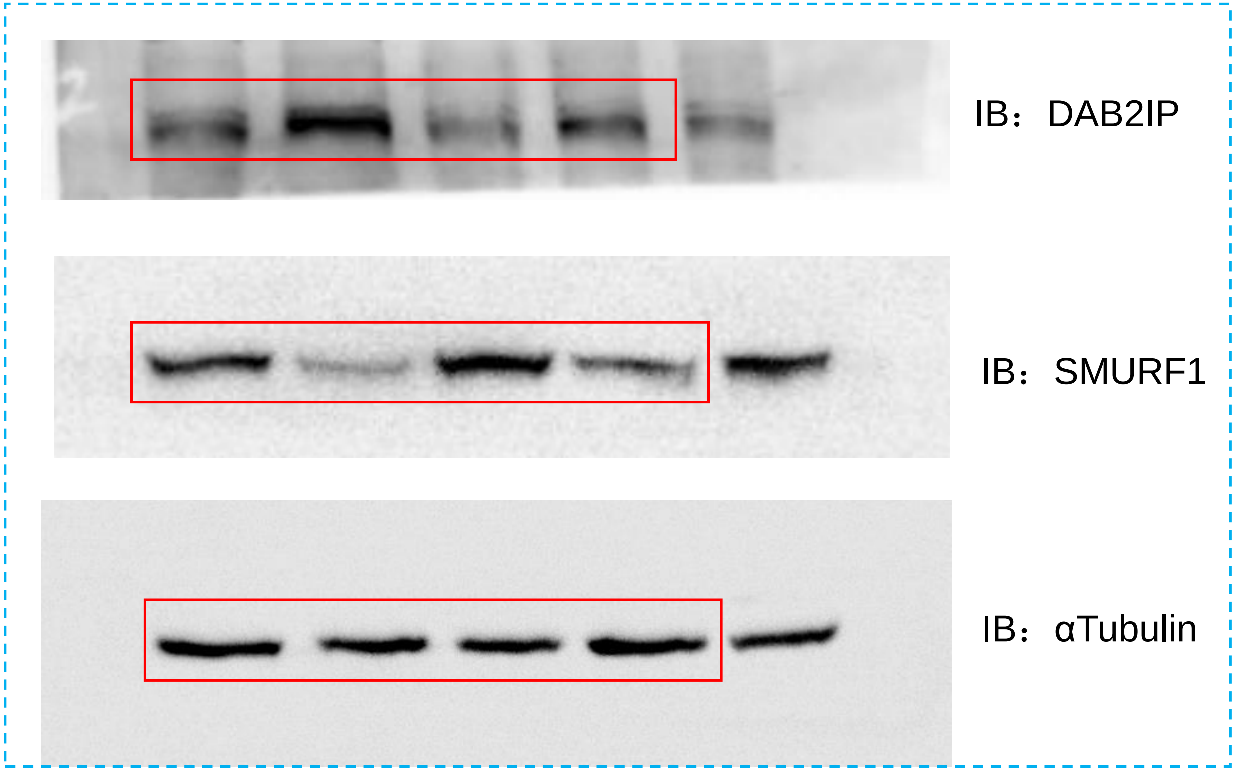

Figure S7C

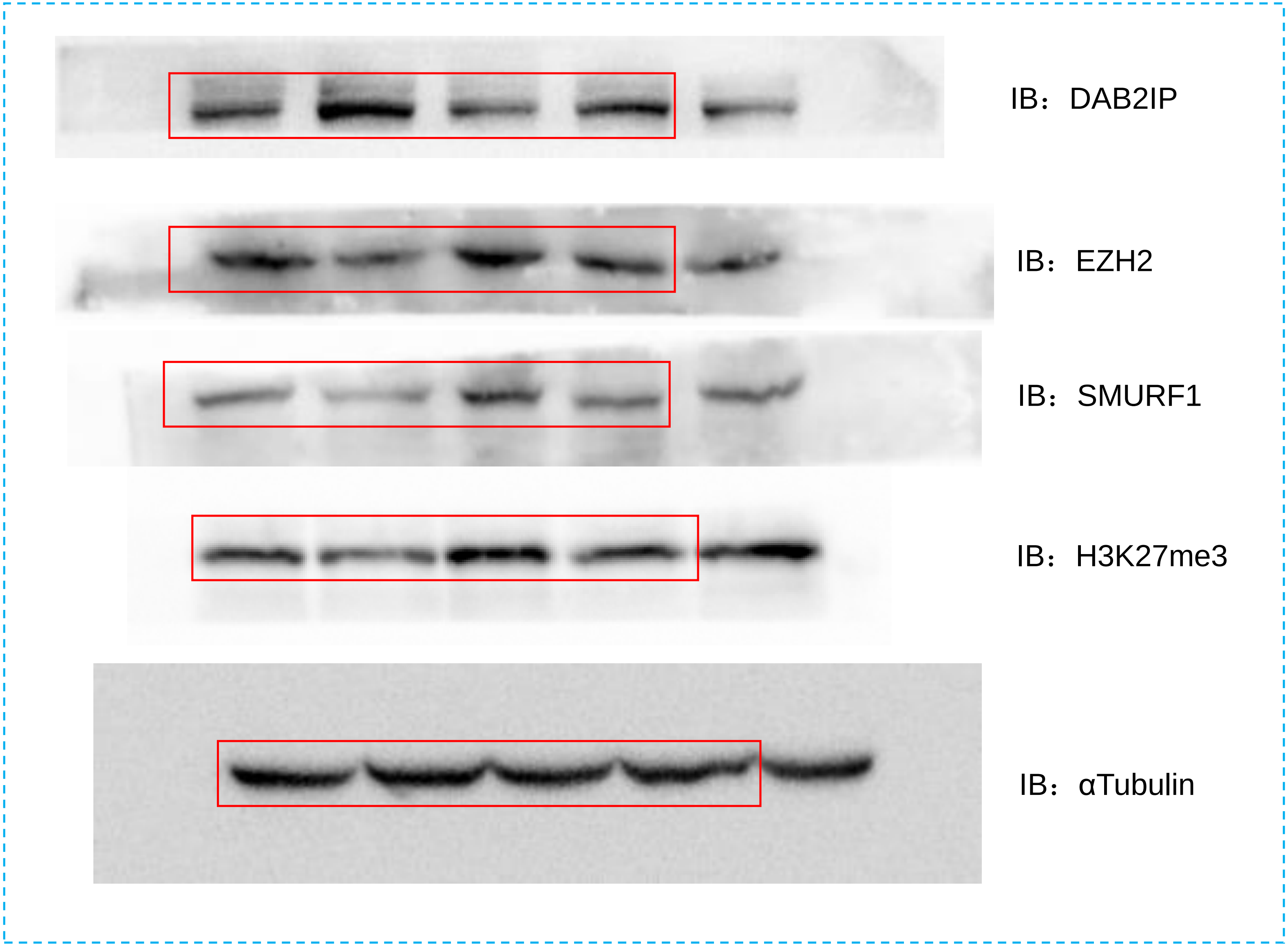

Figure S8A

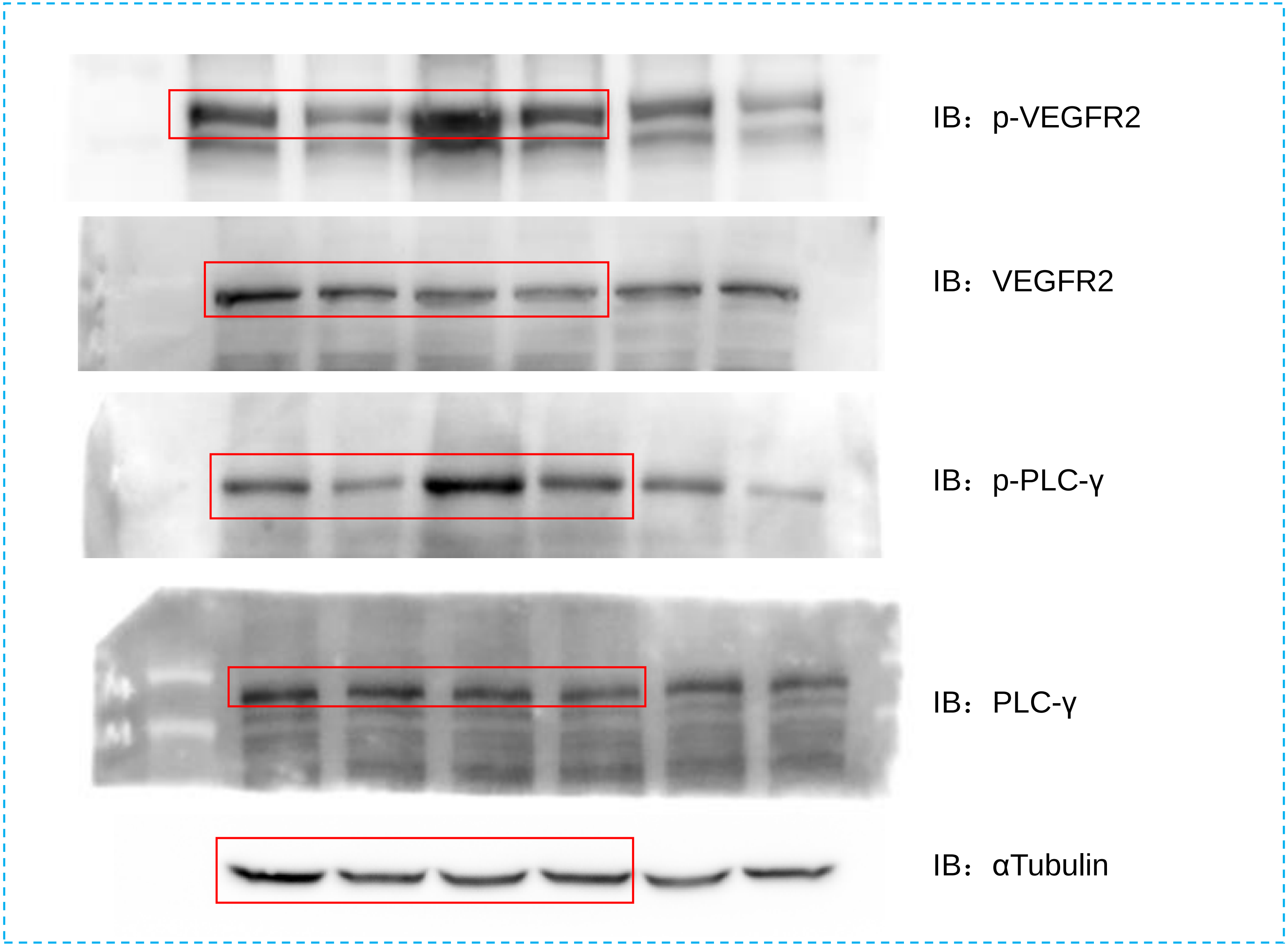

Figure S8B

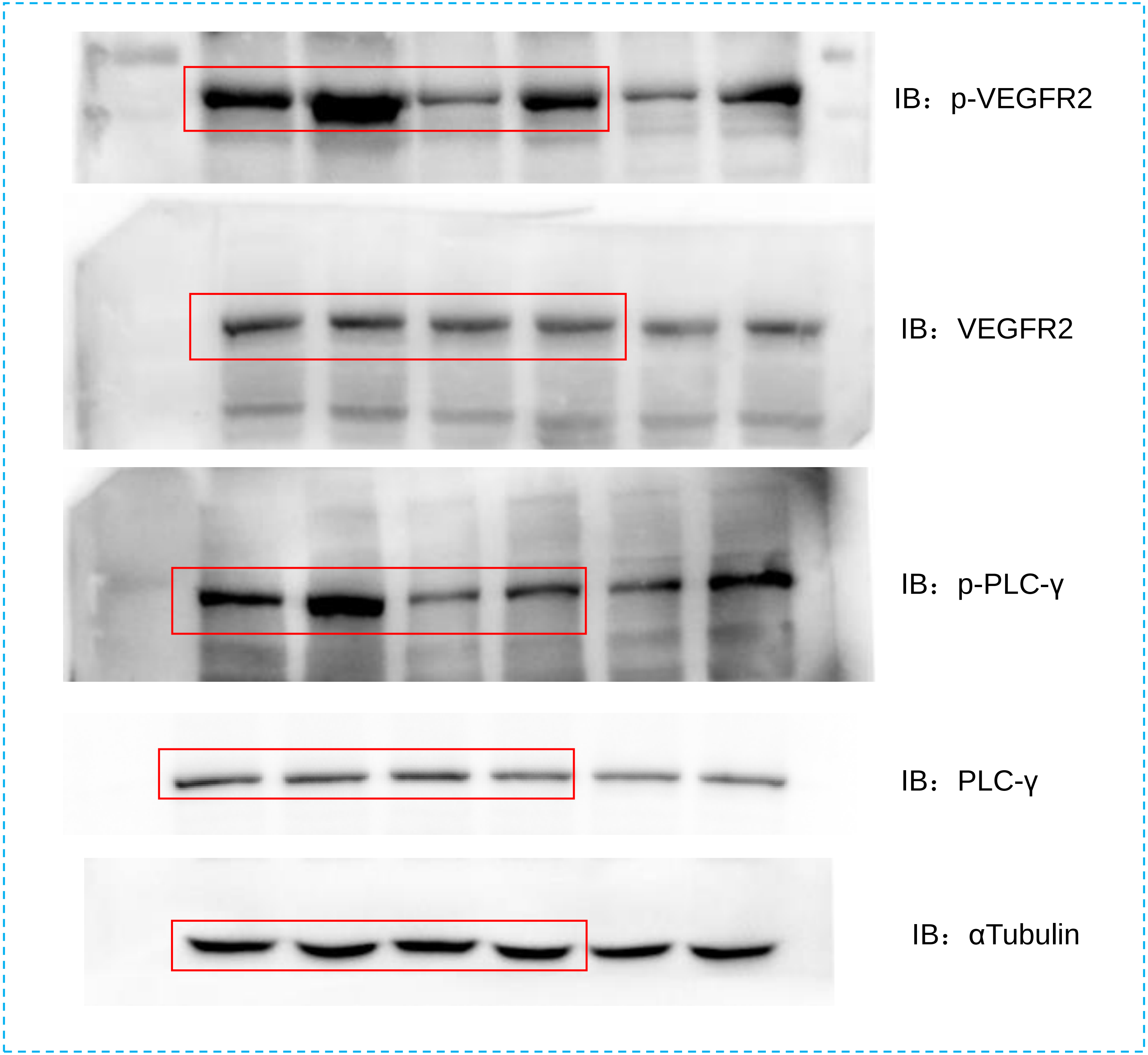

Supplement: Supplementary file 2 — Original western blots [file 41419_2022_4898_MOESM2_ESM.pdf]
